# Effect of Spirulina Meal Supplementation on Growth Performance and Feed Utilization in Fish and Shrimp: A Meta-Analysis

**DOI:** 10.1155/2022/8517733

**Published:** 2022-11-03

**Authors:** Ling Li, Haiyan Liu, Peiyu Zhang

**Affiliations:** ^1^Laboratory of Aquatic Animal Nutrition and Ecology, College of Life Sciences, Hebei Normal University, Shijiazhuang, Hebei 050024, China; ^2^Key Laboratory of Animal Physiology, Biochemistry and Molecular Biology of Hebei Province, Shijiazhuang, Hebei 050024, China

## Abstract

The application potential of spirulina meal in aquaculture feeds has been well summarized in several descriptive reviews. Nevertheless, they converged on compiling results from all possible relevant studies. Little available quantitative analysis regarding the pertinent topics has been reported. This quantitative meta-analysis was performed to investigate the influences of dietary spirulina meal (SPM) addition on responsive variables in aquaculture animals, including final body weight (FBW), specific growth rate (SGR), feed conversion ratio (FCR), protein efficiency ratio (PER), condition factor (CF), and hepatosomatic index (HSI). The pooled standardized mean difference (Hedges' *g*) and 95% confidence limit were computed to quantify the primary outcomes based on random-effects model. The sensitivity and subgroup analyses were carried out to evaluate the validity of the pooled effect size. The meta-regression analysis was conducted to investigate the optimal inclusion of SPM as a feed supplement and the upper threshold of SPM usage for substituting fishmeal in aquaculture animals. The results indicated that on the whole, dietary SPM addition significantly improved FBW, SGR, and PER; statistically decreased FCR of animals; had no significant influence on CF and HSI. The growth-enhancing effect of SPM inclusion in the form of feed additive was significant; however, the effect was indistinctive in the form of feedstuff. Furthermore, the meta-regression analysis displayed that the optimal levels of SPM as a feed supplement in fish and shrimp diets were 1.46%-2.26% and 1.67%, respectively. Additionally, up to 22.03%-24.53% and 14.95%-24.85% of SPM as fishmeal substitute did not have a negative effect on growth and feed utilization in fish and shrimp, respectively. Therefore, SPM is a promising fishmeal substitute and a growth-promoting feed additive for sustainable aquaculture of fish and shrimp.

## 1. Introduction

The global animal protein demand is projected to double by 2050 due to ever increasing human population and growing protein consumption per capita [1]. The amount of protein supplied by global aquaculture accounted for 8% of total animal source protein for human consumption [2], and per capita, consumption is increasing faster than meat and dairy consumption [3]. The aquafeed industry is still dependent on marine ingredients sourced from wild-captured forage fish [4]. Therefore, searching suitable and sustainable alternatives for fishmeal and fish oil is an important approach to achieve continuable growth of aquaculture in production. *Spirulina*, as one of the most extensively used microalgae, has a highly nutritional profile that contains high crude protein content (59%-63% of dry weight), enough n-3 polyunsaturated fatty acids (*γ*-linoleic acid), and vitamins and minerals [5, 6]. Some bioactive substances are also detected in this cyanobacterium, such as phenols, *β*-carotene, chlorophylls, and phycobiliprotein, and they exhibited various biological properties including antioxidant and anti-inflammatory activities [7, 8]. Additionally, recent studies have shown that *Spirulina* could also be cultivated in aquaculture effluent [9, 10], and this is regarded as win-win pattern to treat aquaculture wastewater and spirulina meal production.

In recent years, increasingly studies are accumulating about the application of spirulina meal in fish feed. The spirulina meal was regarded as a functional additive in the diet formulation in some studies, and the supplementation level was comparatively lower. In these studies, they mainly concentrated on growth performance, antioxidative properties [11], immunoprotective effect [12], and pigmentation capability [13, 14] of dietary spirulina meal supplementation for its bioactive compounds. In other studies, spirulina meal was treated as a dietary protein source to substitute fishmeal or other protein sources due to its high protein content and the inclusion level was higher. Considering the nutritional properties of *Spirulina*, practicability analysis of spirulina meal in aquaculture feeds has been well documented in some descriptive reviews [5, 15–20], but they converged on compiling different results from all possible relevant studies. No quantitative reviews regarding the pertinent topics have been reported.

Meta-analysis is a statistically analytical technique to quantitatively combine the results of independent experiments on the same topic, to overall comprehend a problem, determine variation sources, or construct a meta-regression model describing the relationship between variables [21]. Although this analytical technique has been applied in aquaculture nutrition in recent years [22–24], this analytic method has not been widely accepted by the experts due to the short time of application in aquaculture nutrition and the possible difference existed in various cultured species. It is well known that the requirement for dietary nutrients, replacement level of fishmeal by other protein sources or optimal addition level of a dietary additive/ingredient in certain aquaculture species is a range value not a point value under the various aquaculture environment and dietary formulation background. Meta-regression analysis based on the existing data across fish species in the literatures may yield an optimal range of nutrient requirements or substitution levels of fishmeal for fish or shrimps, which could be used for reference to the unstudied cultured species. Given that *Spirulina* is one of the most common microalgae in industrial production, it is necessary to extract data from relevant studies and synthesize the results through meta-analysis. However, there is no systematic review and meta-analysis about the application of spirulina meal in aquafeed across various cultured species. Therefore, the objective of the present study was to conduct meta-analysis to systematically assess the effects of dietary spirulina meal as a functional additive or fishmeal alternative on growth and feed utilization in fish across different studies and species.

## 2. Methods and Materials

### 2.1. Search Strategy

Two researchers established and finalized the literature search strategy. To assess the influence of dietary inclusion of spirulina meal on growth performance (final body weight (FBW), specific growth rate (SGR), feed utilization (feed conversion ratio (FCR), protein efficiency ratio (PER), morphological parameters (condition factor (CF), and hepatosomatic index (HSI) were determined in fish and shrimps. Electronic databases including Web of Science, China National Knowledge Infrastructure (CNKI), and Google Scholar were searched from the inception date until 3^rd^ of March 2022, for the discovery of relevant studies according to Preferred Reporting Items for Systematic Reviews and Meta-Analyses (PRISMA) [25]. The key words and index terms were applied: Topic = (*Spirulina* or *Arthrospira* or *Arthrospira* *maxima* or *Spirulina* *maxima*), Topic = (fish or fishes or shrimp^∗^), and Topic = (diet or diets or dietary or feed or aquafeed^∗^ or aquafeed^∗^). The search was limited to published literatures in English and Chinese and to full-text articles.

### 2.2. Study Selection

#### 2.2.1. Selection of Studies

Citations and abstracts of all retrieved literatures were downloaded to Endnote X9 (for Window, Thomson Reuters, Philadelphia, PA, USA), and duplicates were excluded. Firstly, the titles and abstracts of the searched papers were evaluated by two separate researchers, and off-topic literatures were removed. Secondly, full-text articles of potential studies were downloaded and scrutinized by the same researchers to filter the eligible studies. Thirdly, the third researcher double-checked and finalized the included studies. Lastly, two independent researchers extracted the data from the included studies for meta-analysis. Inconsistencies were resolved by acceptance of a third researcher.

#### 2.2.2. Eligibility Criteria

Articles could be incorporated in this systematic review if they meet the following criteria: (1) the randomized controlled design and procedure were abided by; (2) the experimental animals were fish or shrimps; (3) spirulina meal was supplemented solely in the diet as an additive without large differences in diet nutrient composition or as a fishmeal substitute; (4) at least one of the following parameters was determined in both control and experimental treatments: FBW, SGR, FCR (or feed efficiency), PER, CF, or HSI; (5) mean value, experimental replication, and error estimation (e.g., standard deviation (SD) and standard error (SE)) of each parameter were exhibited either numerically or graphically to compute the effect size and confidence intervals. The studies were excluded when one of the following scenarios existed: (1) the supplemental level of spirulina meal was not clearly indicated; (2) the FM content was not manifested when it was replaced by spirulina meal; (3) animals grew super slowly (SGR was 0.33%/d for 90-day feeding period); (4) the FM was substituted by spirulina meal and other meals; (5) the feeding period was less than 28 days; (6) the experimental replication was one; (7) the extracts of spirulina were used, such as *β*-carotene and phycocyanin.

### 2.3. Data Extraction

A sum of 58 articles were adopted for the current meta-analysis and the following information were gleaned from the result tables in each eligible article: first author's surname; journal; year published; study design including cultured species, initial body weight, number of replicates, number of animals per replicate, and feeding period of the trial; culture condition containing salinity and water temperature; contents of spirulina meal (SPM) and fishmeal (FM) in control and experimental diets; outcome measurements (FBW, SGR, FCR, PER, CF, and HSI), and homologous SD, SE, or pooled SE. When the results of the target variables were presented in the form of a graph, Origin 8.5 software was implemented to extract data. Moreover, the trophic levels of fish and shrimp were acquired from FishBase (http://www.fishbase.se/) and earlier studies [26–28]. One author extracted data from each study and another author proofread all data entries for accuracy. In several studies, feed efficiency (FE), but not the FCR, was used to evaluate the feed utilization of animals. The mean and error of FE were transformed to the counterparts of FCR in accordance with our previous study [29], and the equations were as follows: SD_FCR_ = SD_FE_/(Mean_FE_ × Mean_FE_); Mean_FCR_ = 1/Mean_FE_.

The equations of growth parameters in the included studies were shown as follows:

1. Specific growth rate (SGR, %/d) = 100 × [ln (FBW) − ln (IBW)]/days
2. Feed conversion ratio (FCR) = feed intake/(final body weight − initial body weight)
3. Protein efficiency ratio (PER, %) = 100 × body weight gain/protein intake
4. Condition factor (CF, %) = 100 × final body weight of each fish or shrimp (*g*)/(final body length of each fish or shrimp)^3^
5. Hepatosomatic index (HSI, %) = 100 × final liver weight of each fish (*g*) or final hepatopancreas weight of each shrimp/final weight of each fish or shrimp (*g*)

### 2.4. Data Synthesis and Analysis

All statistical analyses and graphical approaches were performed in the R version 4.0.2 platform. The metafor package was used to calculate effect size, sampling variation, and 95% confidence interval (95% CI) and to analyze heterogeneity and sensitivity. The segmented package was implemented to judge the breakpoint in the broken-line regression. The ggplot2 package was applied to construct the forest plots and regression plots.

#### 2.4.1. Effect Size Computation and Heterogeneity Analysis

Standardized mean difference (SMD) effect size was expressed by Hedges' *g* statistic and the computational formula referred to our previous study [29]. In some studies, standard error (SE) or pooled standard error (PSE) were used to assess within-group variation. The SE or PSE was transformed to standard deviation (SD) through the following equation: SD = SE × sqrt (*n*), where *n* is the number of study replicates. The Hedges' *g* effect size and corresponding 95% CI were computed by a random-effects model for each outcome indicator (FBW, SGR, FCR, PER, CF, and HSI) considering that the outcome indicators were continuous variables. A positive Hedges' *g* value (lower 95% CI>0) indicates that the outcome indicator of fish or shrimp was significantly higher in SPM supplemented group compared with SPM free group. A negative Hedges' *g* value (upper 95% CI<0) represents that the outcome indicator was significantly lower in spirulina meal supplemented treatment compared with spirulina free treatment. The 95% CI of Hedges' *g* estimate encompassing zero denotes that no significant variations of the indicators were observed between control group and experimental group. The Hedges' *g* values could also be explained as follows: small effect (*g* = 0.20 − 0.49), medium effect (*g* = 0.50 − 0.79), and large effect (*g* = 0.80 and above). Cochran's *Q* statistic and *I*^2^ statistic were implemented to quantitatively categorize heterogeneity across studies as follows: no heterogeneity: 0 < *I*^2^ ≤ 25%; low heterogeneity: 25% < *I*^2^ ≤ 50%; moderate heterogeneity: 50% < *I*^2^ ≤ 75%; high heterogeneity ≥75%.

#### 2.4.2. Sensitivity Analysis and Publication Bias

Sensitivity analysis was performed to identify outliers and influential data points by using the Cook's *D* distance and *R*-Student value. Once being identified as influential points, these data were removed from the dataset, and the overall Hedge's *g*, CI, and *I*^2^ were reanalyzed. The publication bias was evaluated by constructing funnel plots. Moreover, the trim and fill method was conducted to assess missing studies and modify the overall effect size. Begg's and Egger's tests were also used, and the statistical level was set at *p* < 0.1 [30]. If the results of Begg's and Egger's tests were incompatible, the Egger's test was adopted as a reference.

#### 2.4.3. Subgroup Analysis and Meta-regression Analysis

Effects of experimental species, SPM as a feed additive or feedstuff, on trophic levels of aquatic animals were tested by subgroup analysis. We classified the subgroups in three groups: (1) the experimental animals were categorized as fish and shrimp; (2) the fish was further subdivided into freshwater fish and marine fish according to culture environment; (3) SPM was defined as a feed additive when the inclusion level in the diet was less than 4% (≤4%) and as a feed ingredient when the inclusion level was more than 4% (>4%); (4) the rank of trophic level of animals was defined as follows according to earlier study: low trophic level: 2 ≤ trophic level<3, medium trophic level: 3 ≤ trophic level<4, and high trophic level: trophic level ≥4 [29].

In view of the inclusion level of SPM being a continuous variable, the random-effects meta-regression analysis was conducted to probe into the regression relationship between the inclusion content of SPM and the effect size of each outcome indicator. The datasets of FBW, SGR, FCR, CF, and HSI were divided into two subsets according to use of spirulina meal as a feed additive or ingredient, respectively. These subsets were then subdivided into two smaller subsets based on the experimental animals being fish or shrimp. When the SPM was viewed as additive, the optimal levels of SPM were estimated through the fitted regression equations. In addition, the superior limits of SPM usage as a protein ingredient were assessed by the intersection point of the fittest regression curve and Hedge's *g* at zero. The datasets of PER, CF, and HSI were not metaregressed due to relatively smaller sample capacity. Furthermore, local polynomial regression was conducted to determine the tendency between spirulina meal contents and Hedge's *g* values using the ‘loess' method of geom_smooth function in the ggplot2 package when exploring the best fitted curve. Subsequently, linear, quadratic, cubic, and broken-line regressions were chosen to fit the data, and the best fitted curve/equation was selected. In addition, we used the segmented function in the segmented package to determine the breakpoint and piecewise equations in the broken-line regression.

## 3. Results

### 3.1. Study Selection Process

**Figure 1:**
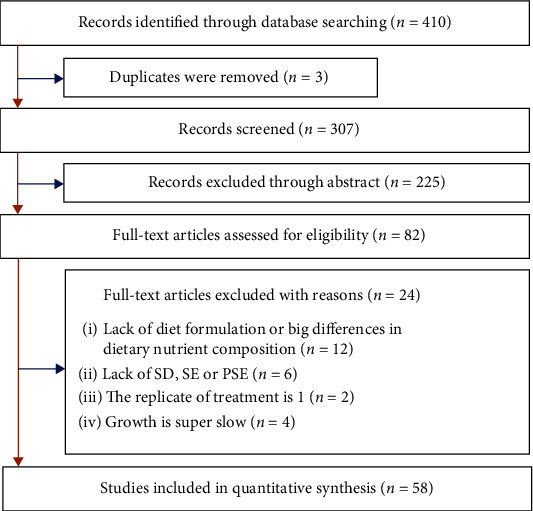
Flow diagram of eligible study selection according to Preferred Reporting Items for Systematic Reviews and Meta-Analyses (PRISMA). The SD, SE, and PSE represented standard deviation, standard error, and pooled standard error, respectively.

The study selection process was detailed in Figure 1. The initial literature search yielded 410 publications, of which three repeated articles were omitted and 100 were excluded after reviewing the titles and 225 based on abstract assessment. Full texts of 82 probably eligible publications were retrieved for further evaluation, of which 24 articles were removed for the following reasons. Twelve articles lacked of diet formulas or big differences existed in the nutrient composition of experimental diets; six studies did not indicate the error values (standard deviation, standard error, or pooled standard error) of the parameters; one article only had one replicate; four articles had super slow growth rate. After filtration, 58 articles were finally included in this review.

### 3.2. Characteristics of Included Studies

Information of the included articles were recapitulated in Table S1 of Supplemental Materials. The publication year of the incorporated studies ranged from 2005 to 2022. Twenty-eight species were encompassed in the study. Six shrimp species were studied, including *Fenneropenaeus chinensis* [31], *Litopenaeus vannamei* ([32–34], and [35]), *Macrobrachium rosenbergii* [36], *Marsupenaeus japonicus* [37], *Neocaridina davidi* [38], *Penaeus monodon* ([39, 40], and [41]), and 22 fish species included *Amphilophus citrinellus* × *Cichlasoma trimaculatum* [42], *Astronotus ocellatus* [12], *Barilius bendelisis* [43], *Carassius auratus* [44], *Carassius auratus gibelio* [45–47], *Clarias gariepinus* [48, 49], *Clarias macrocephalus* [50], *Cyprinus carpio* ([51–53], and [13, 14]), *Cyrtocara moorii* [54, 55], *Dicentrarchus labrax* [56], *Megalobrama amblycephala* [57], *Mugil liza* [17–20], *Oncorhynchus mykiss* ([58–61], and [62]), *Oplegnathus fasciatus* [63], *Oreochromis niloticus* ([64–77], and [78]), *Pagrus pagrus* [79], *Pangasinodon gigas* [80], *Pelteobagrus fulvidraco* ([81–83], and [11]), *Piaractus mesopotamicus* [84], *Salmo trutta caspius* [85, 86], *Solea solea* [87, 88], and *Trichopodus trichopterus* [89]. Among the 28 species, 11 belonged to low trophic species, 16 were medium trophic species, and 1 was high trophic species (*O. mykiss*). The dietary content of SPM as feed additives in the experimental treatment scoped from 0.025% to 4%, and the extent of SPM as feed ingredients was from 4.5% to 59.7%. A sum of 620 comparisons (*n* = 148 for FBW, *n* = 149 for SGR, *n* = 147 for FCR, *n* = 77 for PER, *n* = 65 for CF, and *n* = 34 for HSI) between the control group and spirulina meal supplemented group were carried out in this study (Tables 1 and 2 and Table S2). Considering that dietary spirulina meal supplementation generated no impact on morphological indices (CF and HSI), the meta-analyzed results were placed in Tables S2, S7, and S8 and Figures S5, S6, and S7 in Supplemental Materials.

### 3.3. Effect of Dietary SPM Addition on FBW

**Figure 2:**
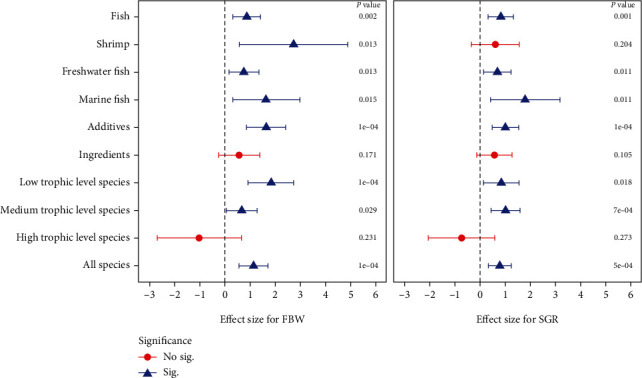
Hedges' *g* comparisons for FBW and SGR (mean ± 95% confidence interval) subgroup analysis (random-effects model). The confidence interval intersecting with the dashed line indicated no significant differences between the control group and treatment group, and vice versa.

A total of 148 comparisons were executed to investigate the overall effect of dietary SPM inclusion on FBW of aquatic animals (Table 1), and the results of publication bias for FBW were shown in Table S3 and Figure S1. The results suggested that dietary SPM addition significantly improved FBW compared to control group (Hedges' *g* = 1.14, 95%CI = 0.57 to 1.71, *P* < 0.0001) with big heterogeneity (*I*^2^ = 99.09%, *P*_*heterogeneity*_ < 0.0001). Subgroup analysis was carried out in this study to explore the sources of such high heterogeneity. According to possible factors, the FBW dataset was decomposed based on experimental animals, habitat of cultured fish, usage of spirulina meal, and trophic level (Table 1 and Figure 2). The subgroup analysis displayed that dietary supplementation of spirulina meal significantly improved FBW in fish (Hedge's *g* = 0.87, *P* = 0.002), freshwater fish (*g* = 0.75, *P* = 0.013), marine fish (*g* = 1.65, *P* = 0.015), shrimp (*g* = 2.73, *P* = 0.013), additives subgroup (*g* = 1.65, *P* < 0.0001), low trophic level species (*g* = 1.83, *P* < 0.0001), and medium trophic level species (*g* = 0.67, *P* = 0.029). Whereas, species with high trophic levels (*g* = −1.02, *P* = 0.23) and a subgroup of ingredients (*g* = 0.57, *P* = 0.17) were not significantly affected by dietary addition of SPM.

**Figure 3:**
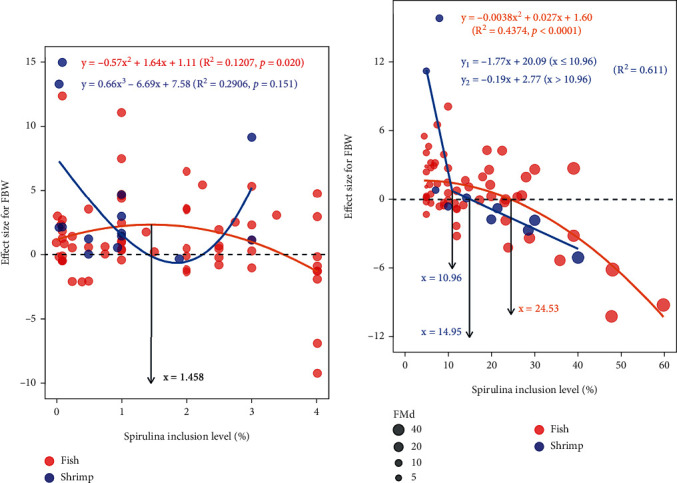
Random-effects model regression of spirulina meal inclusion level as a feed additive (a) and feed ingredient (b) on the effect size of FBW. The circle size represents the replaced level (%) of fishmeal by SPM, in other words, it means the difference of fishmeal content between the control group and SPM supplemented group.

Moreover, meta-regression was also performed to explore the possible relationship between the effect size of FBW (EF_FBW_) and SPM inclusion level as a feed additive or a feed ingredient in fish and shrimp. As a feed additive, a significantly quadratic relationship between EF_FBW_ and SPM inclusion level in fish was observed (*P* = 0.02, *R*^2^ = 0.121), and optimal level of *Spirulina* was estimated to be 1.46% through the fitted equation (Figure 3(a)). As a feed ingredient, the best fittest curve was also a quadratic equation for fish (*P* < 0.0001, *R*^2^ = 0.437), and the upper limit of SPM level in fish diet was calculated to be 24.53%, exceeding which negative influence of SPM on FBW of fish would emerge (Figure 3(b)). As for shrimp, the broken-line regression was the better fitted one, and the biggest usage of spirulina meal in the diet was estimated as 14.95% (Figure 3(b)).

### 3.4. Effect of Dietary SPM Addition on SGR

A sum of 149 comparisons for examining the influence of dietary SPM addition on SGR in aquaculture animals were shown in Table 1, and the results of publication bias for SGR were exhibited in Table S4 and Figure S2. The results indicated that dietary SPM supplementation had a significantly beneficial effect on SGR in all species (Hedges' *g* = 0.79, 95%CI = 0.34 to 1.23, *P* = 0.001) with high heterogeneity (*I*^2^ = 98.69%, *P*_*heterogeneity*_ < 0.0001). In the subgroup analysis, the overall trend of EF_SGR_ in each subgroup was exactly alike as to those of EF_FBW_ except for the shrimp subgroup. In this subgroup, the effect of SPM on SGR in shrimp showed no significant difference (*g* = 0.61, 95%CI = −0.33 to 1.56, *P* = 0.204).

**Figure 4:**
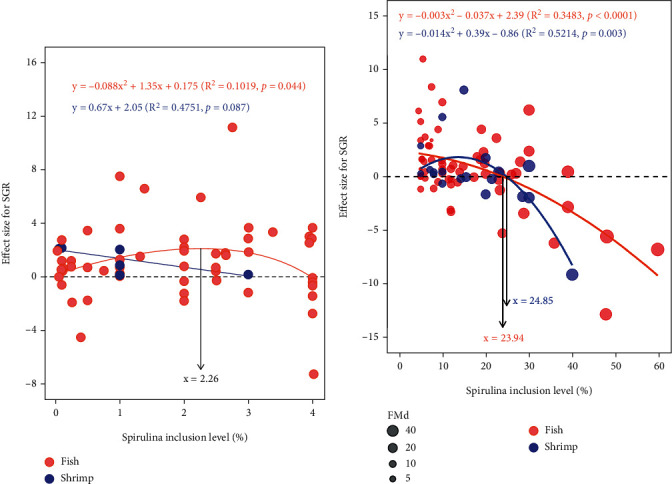
Random-effects model regression of spirulina meal inclusion level as a feed additive (a) and feed ingredient (b) on the effect size of SGR. The circle size represents the replaced level (%) of fishmeal by SPM, in other words, it means the difference of fishmeal content between the control group and SPM supplemented group.

As a feed additive, SPM inclusion level showed a significantly cubic relationship with EF_SGR_ in fish (*P* = 0.04, *R*^2^ = 0.102), and optimum level of SPM based on cubic equation was recommended to be 2.26% (Figure 4(a)). As a feed ingredient, quadratic regression was the best fitted curve both in fish and shrimp, and the highest usage of SPM in fish and shrimp were estimated to be 23.94% and 24.85%, respectively (Figure 4(b)).

### 3.5. Effect of Dietary SPM Addition on FCR

**Figure 5:**
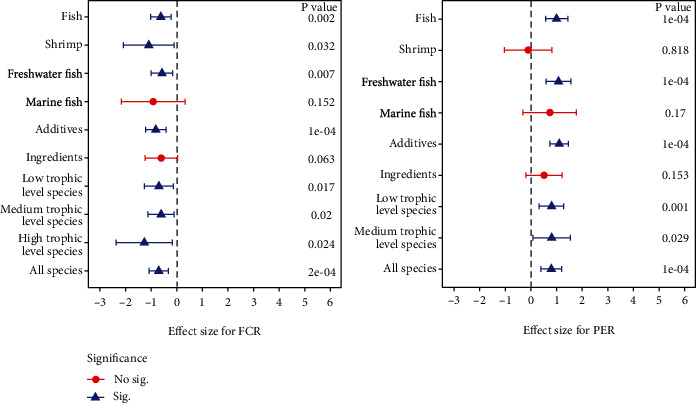
Hedges' *g* comparisons for FCR and PER (mean ± 95% CI) subgroup analysis (random-effects model). The confidence interval intersecting with the dashed line indicated no significant differences between the control group and treatment group, and vice versa.

A sum of 147 comparisons were carried out to investigate the influence of dietary administration of SPM on FCR in aquaculture animals (Table 2 and Figure 5), and the results of publication bias for FCR were exhibited in Table S5 and Figure S3. We found that SPM included in diets clearly decreased FCR of fish and shrimp (Hedges' *g* = −0.70, 95%CI = −1.07 to − 0.33, *P* = 0.0002). The subgroup analysis manifested that dietary spirulina addition noteworthily reduced FCR in all subgroups excepting the subgroups of ingredients and marine fish, in which there were no obvious differences in FCR between the control group and SPM supplemented group (*g* = −0.60, *P* = 0.06 for ingredients group; *g* = −0.91, *P* = 0.15 for marine fish group) (Table 2 and Figure 5).

**Figure 6:**
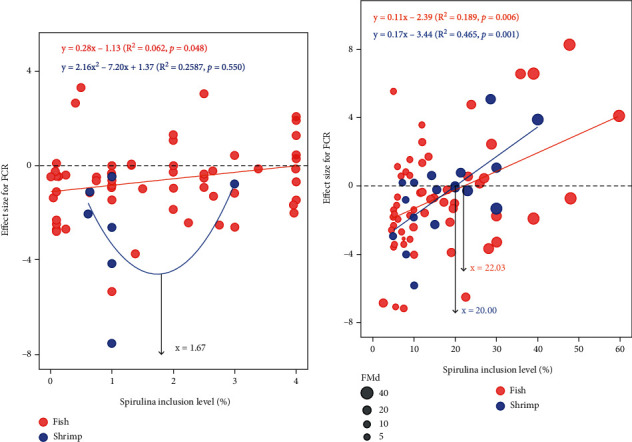
Random-effects model regression of spirulina meal inclusion level as a feed additive (a) and feed ingredient (b) on the effect size of FCR. The circle size represents the replaced level (%) of fishmeal by SPM, in other words, it means the difference of fishmeal content between the control group and SPM supplemented group.

In the meta-regression, there was a significantly negatively linear correlation between the effect size of FCR (EF_FCR_) and spirulina level in fish diet (*P* = 0.048, *R*^2^ = 0.062) (Figure 6(a)) when spirulina was treated as a feed additive. Optimum level of SPM in shrimp diet based on the quadratic equation was advised to be 1.67% (Figure 6(a)). As a feedstuff, linear regression was the best fitted curve both in fish and shrimp, and the upper limits of spirulina meal usage in fish and shrimp were calculated separately to be 22.03% and 20.00% (Figure 6(b)).

### 3.6. Effect of Dietary SPM Addition on PER

A sum of 77 comparisons were conducted to examine the effect of dietary addition of SPM on PER in aquaculture animals (Table 2 and Figure 5), and the results of publication bias for PER were exhibited in Table S6 and Figure S4. We discovered that dietary addition of SPM significantly improved PER in fish and shrimp (Hedges' *g* = 0.80, 95%CI = 0.39 to 1.20, *P* = 0.0001) with high heterogeneity (*I*^2^ = 96.69%, *P*_*heterogeneity*_ < 0.0001). The subgroup analysis manifested that dietary SPM addition clearly increased PER in all subgroups excepting shrimp, marine fish, and ingredient subgroups. There were no obvious variations in PER for shrimp subgroup (*g* = −0.11, *P* = 0.82), marine fish group (*g* = 0.73, *P* = 0.17), and ingredient subgroup (*g* = 0.51, *P* = 0.15) between control group and SPM supplemented group (Table 2 and Figure 5). Due to the small sample size, meta-regression analysis between effect size of PER and SPM inclusion levels in fish and shrimp was not performed.

## 4. Discussion

Spirulina meal (SPM) has garnered increasing attention in the past few years and has been extensively studied as a novel protein source and a functional feed additive in aquafeeds. There existed big differences in the application efficacy of SPM in different aquaculture animals when it was regarded as a protein source in the diet formula. Fish meal could be 100% substituted by SPM with higher growth rate and better feed utilization in tilapia [69], while in *C. moorii* and *A. citrinellus* × *C. trimaculatum*, a small quantity of replacement for FM resulted in inferior growth and feed utilization [42, 54, 55]. Likewise, SPM, as a functional additive, exerted a negative influence on growth performance in some studies [82]. Whereas, high potency has been observed in rainbow trout when the supplemental dose of SPM was optimal [61]. The efficacy instability of SPM was also well reflected in the current study, in which large confidence intervals of overall effect size were noticed in FBW (0.51 to 1.71), SGR (0.34 to 1.23), FCR (-1.07 to -0.33), and PER (0.39 to 1.20). In spite of the high heterogeneity, the immutable fact was that the mean effect sizes of FBW, SGR, FCR, and PER was favorable for animals and were 1.14, 0.79, -0.70, and 0.80, respectively. Meanwhile, the average effect sizes of CF and HSI were not significantly affected by SPM administration. It is indicative that on average dietary, SPM inclusion significantly improved growth performance and feed utilization of aquaculture animals.

Although the pooled effect sizes of outcome parameters on the whole dataset validated the benefits of SPM on growth performance, the degree of a cross-study heterogeneity was large and this meta-analysis clearly averaged out some key influential factors, including cultured species (fish and shrimp), use of SPM (ingredient and additive), trophic levels of animals (low, medium, and high), and inclusion level of SPM. The primary target of a subgroup analysis is to recognize either consistency or big variances in the magnitude of the treatment effect among different groupings [90]. Therefore, the subgroup analysis was employed to characterize the effects of some of the aforementioned main covariates. Meta-regression aims at distinguishing whether a linear relationship exists between an outcome measure and a continuous variable [91]. Thus, the meta-regression analysis was conducted to explore the most probable relationship between the pooled effect size of each parameter and SPM inclusion level. We could determine the optimal concentration of SPM in the diet as a feed additive by means of meta-regression analysis and identify the upper threshold of SPM substituting fishmeal in diet beyond which the negative effects on growth might occur.

The subgroup analysis revealed that dietary SPM addition significantly improved growth and feed utilization in fish with large sample sizes (*k* = 124 for FBW, 123 for SGR, 121 for FCR, and 63 for PER). From the mathematical point of view, the majority of Hedge's *g* from the fish subsets of FBW, SGR, and PER are distributed at more than zero (75 in FBW, 91 in SGR, and 53 in PER). Meanwhile, most of data in the FCR subset distributed at less than zero (91 in FCR). Therefore, the overall synthesized results of the growth of fish fed SPM were beneficial for fish. The SPM was applied in fish diet in the forms of a feed supplement and a FM substitute. When the SPM was included in the fish diet as a supplement, the growth-promoting effects might be attributed to its high nutrient intensity (high protein content, *γ*-linolenic acid, etc.) and bioactive compounds (polysaccharides, carotenoids, chlorophyll, etc.) [17–20]. This could also be mirrored in the regression charts, in which most of the red dots dispersed above the dashed line (*g* = 0) for FBW and SGR subgrouping (Figures 3(a) and 4(a)). For FCR subsets, the red dots scattered mainly below the reference line (Figure 6(a)). When the SPM was used in the form of a feed ingredient, low and medium substitution levels of fishmeal (not exceeding a certain replacement levels) benefiting the growth of fish compared to the control diets were recorded in many studies [17–20, 36, 42, 46, 47, 60, 74]. Apart from the active components in SPM, this growth-promoting effect may be also correlated with the modified composition of gut microbiota by SPM inclusion in fish diets. It has been reported that adding 15% SPM in zebrafish diet significantly increased the abundance of beneficial bacteria in the gut such as *Cetobacterium* and decreased the proportion of pernicious bacteria such as *Vibrio* [92]. *Cetobacterium* is one of the most common bacteria in the intestine of freshwater fish and could produce vitamin B_12_, which is essential nutrient for fish [93], and *Vibrio* is one of the most prevalent pathogens and leads to vibriosis in fish [94]. Similarly, SPM has also been found to alter the intestinal microbiota structure in Yellow River carp and exert a positive influence on health [13, 14]. Regarding the shrimp subgroup, SPM supplementation significantly altered FBW and FCR, but had no significant differences on SGR and PER. This may be due to the small sample size in this dataset (*k* = 24 for FBW, 26 for SGR, 26 for FCR, and 14 for PER).

In terms of additive subgroups, SPM addition noteworthily enhanced growth performance in fish and shrimp, which could be ascribed to its easy digestibility [95], and abundant antioxidants, including phycocyanin, polysaccharide, polyunsaturated fatty acids, vitamins, carotenoids, and other bioactive compounds [96]. It was because of the strong antioxidation properties of the *Spirulina*, some energy for metabolic processes could be saved for animal growth. Whereas in the ingredient subgroup, SPM inclusion had no significant effect on animal growth. This may be attributed to the negative impact of high substitution levels of fishmeal by SPM in some studies [74, 83]. We also conducted the subgroup analysis based on the trophic level of an animal. It has been found that SPM supplementation significantly affected FBW, SGR, FCR, and PER in low and medium trophic species. In the high trophic species (O. *mykiss*) subgroup, there were no significant variances in FBW and SGR between the control group and SPM group. It could be correlated with the feeding habit of the animal, low and medium trophic animals belonging to herbivores or omnivore, they could digest some microalgae well in the gut in the natural waters from an evolutionary point of view. However, *O. mykiss* appertains to carnivores, the effect of dietary SPM as a supplement or a feed ingredient on growth was poor in this fish [58, 61, 62].

To investigate the optimal inclusion of SPM as a feed supplement and the superior limit of SPM inclusion level for substituting fishmeal in fish and shrimp, the meta-regression between the effect sizes of outcome measures (FBW, SGR, and PER) and SPM inclusion level was carried out. It indicated that the optimal addition level of SPM in fish diet was estimated to be 1.46%-2.26% based on the effect sizes of FBW and SGR with SPM inclusion level. In addition, the lowest value of FCR effect size was assessed at 1.67% of SPM inclusion level in shrimp. The upper limit of SPM inclusion level replacing fishmeal ranged from 22.03% to 24.53% in fish. This scope was agreed with previous studies, in which the maximal usage of SPM meal was estimated at 23.03% in *P. fulvidraco* [83] and 22.50% in *M. liza* [17–20] from the viewpoint of growth. Whereas this extent of SPM was broader from 14.95% to 24.85% in shrimp, this may be related to the small sample size in shrimp subsets. This also may be due to different shrimp species in the FBW subset and SGR subset. There were three shrimp species in the FBW subset, including *L. vannamei* [32], *N. davidi* [38], and *M. rosenbergii* [36], whereas the SGR subclass had another species (*P. monodon*). This shrimp species, with an omnivorous feeding habit, was demonstrated to have the capability to utilize at least 20.00% SPM in the diet without jeopardizing growth performance [40]. Therefore, the maximum usage of SPM in shrimp diet reached 24.85%.

## 5. Conclusion

In a nutshell, the present quantitative meta-analysis indicated that dietary SPM addition significantly improved final body weight, specific growth rate, and protein efficiency ratio, and simultaneously significantly decreased the feed conversion ratio of aquaculture animals. In addition, the growth-enhancing effect of SPM inclusion was significant when it was applied in the form of feed additive (the inclusion level was less than 4%); however, the effect was indistinctive when it was utilized as a feedstuff. Furthermore, the meta-regression analysis displayed that the optimal addition levels of SPM as a growth-promoting feed supplement in fish and shrimp diets were 1.46%-2.26% and 1.67%, respectively. Furthermore, application of SPM in diet could efficiently regulate the body color in fish according previous studies. Therefore, despite high price of SPM, it could be also applied as a diet additive currently for aquatic animals having higher demand for body color in the market, such as *P. fulvidraco* and some ornamental fish. Additionally, up to 22.03%-24.53% and 14.95%-24.85% of SPM as fishmeal substitute in fish and shrimp diets did not have a negative effect on growth and feed utilization, respectively. Due to high price of SPM, it could be put into practice in aquafeeds as main protein sources in the future when the market price declines sharply.

## Figures and Tables

**Table 1:** Effect size calculation for final body weight (FBW) and specific growth rate (SGR) comparison between control treatment and spirulina meal supplemented treatment across aquaculture species through random-effects model.

	Effect size (random-effects model) for FBW						Effect size (random-effects model) for SGR					
	*k*	*I* ^2^	Hedges' *g* value	SE	CL	*P* value	*k*	*I* ^2^	Hedges' *g* value	SE	CL	*P* value
All species	148	99.09	1.141	0.290	0.572 to 1.709	<0.0001	149	98.69	0.788	0.227	0.343 to 1.234	0.001
Subgroups												
Species category												
Fish species	124	98.66	0.871	0.279	0.323 to 1.419	0.002	123	98.59	0.829	0.257	0.326 to 1.332	0.001
Shrimps	24	99.75	2.731	1.100	0.580 to 4.882	0.013	26	98.84	0.614	0.483	-0.334 to 1.561	0.204
Fish + habitat												
Freshwater fish	108	98.54	0.754	0.305	0.157 to 1.350	0.013	108	98.26	0.694	0.274	0.156 to 1.232	0.011
Marine fish	16	98.42	1.645	0.679	0.314 to 2.976	0.015	15	98.64	1.795	0.702	0.420 to 3.170	0.011
Supplemental types												
Additives	78	98.86	1.645	0.396	0.870 to 2.420	<0.0001	68	97.16	1.016	0.265	0.496 to 1.536	0.0001
Ingredients	70	99.20	0.572	0.418	-0.247 to 1.391	0.171	81	99.17	0.580	0.358	-0.122 to 1.281	0.105
Trophic level												
Low trophic level	80	99.12	1.831	0.465	0.920 to 2.742	<0.0001	79	98.55	0.845	0.357	0.146 to 1.544	0.018
Medium trophic level	54	98.28	0.671	0.307	0.069 to 1.272	0.029	58	98.52	1.018	0.300	0.430 to 1.605	0.001
High trophic level (*O. mykiss*)	14	98.73	-1.019	0.852	-2.688 to 0.650	0.231	12	97.90	-0.737	0.671	-2.053 to 0.579	0.273

*k*: sample size (no. of comparison); *I*^2^: percentage variation across studies due to heterogeneity; SE: standard error; CL: confidence limits (lower and upper).

**Table 2:** Effect size calculation for feed conversion ratio (FCR) and protein efficiency ratio (PER) comparison between control treatment and spirulina meal supplemented treatment across aquaculture species through random-effects model.

	Effect size (random-effects model) for FCR						Effect size (random-effects model) for PER					
	*k*	*I* ^2^	Hedges' *g* value	SE	CL	*P* value	*k*	*I* ^2^	Hedges' *g* value	SE	CL	*P* value
All species	147	97.71	-0.700	0.190	-1.072 to -0.328	0.0002	77	96.69	0.796	0.207	0.390 to 1.202	0.0001
Subgroups												
Species category												
Fish	121	97.29	-0.615	0.202	-1.011 to -0.218	0.002	63	95.94	1.003	0.224	0.565 to 1.442	<0.0001
Shrimp	26	98.73	-1.089	0.508	-2.085 to -0.093	0.032	14	97.82	-0.109	0.471	-1.032 to 0.815	0.818
Fish + habitat												
Freshwater fish	107	97.31	-0.575	0.214	-0.995 to -0.155	0.007	49	95.90	1.076	0.248	0.591 to 1.561	<0.0001
Marine fish	14	97.09	-0.910	0.635	-2.155 to 0.334	0.152	14	96.06	0.728	0.531	-0.313 to 1.768	0.170
Supplemental types												
Additives	70	96.06	-0.810	0.203	-1.207 to -0.413	<0.0001	36	91.52	1.100	0.181	0.745 to 1.454	<0.0001
Ingredients	77	98.33	-0.596	0.321	-1.224 to 0.032	0.063	41	97.62	0.513	0.358	-0.190 to 1.215	0.153
Trophic level												
Low trophic level	81	98.06	-0.691	0.289	-1.258 to -0.124	0.017	45	95.70	0.801	0.243	0.325 to 1.277	0.001
Medium trophic level	57	97.33	-0.606	0.261	-1.118 to -0.094	0.020	32	97.59	0.813	0.372	0.083 to 1.542	0.029
High trophic level (*O. mykiss*)	9	91.10	-1.261	0.557	-2.352 to -0.170	0.024	—	—	—	—	—	—

*k*: sample size (no. of comparison); *I*^2^: percentage variation across studies due to heterogeneity; SE: standard error; CL: confidence limits (lower and upper).

## Data Availability

Data are available on request from the authors.

## Supplementary Materials

Supplementary Materials The detailed information of included studies in this meta-analysis was listed in Table S1 of Supplemental Materials. The meta-analysis for CF and HSI were presented in Table S2 and Figure S7. In addition, publication bias for each outcome indicator (FBW, SGR, FCR, PER, CF, and HSI) in this study evaluated by methods of Egger's regression test, Begg's rank correlation test, and funnel plot were shown in Tables S3-S8 and Figures S1-S6, respectively. Furthermore, R code in this meta-analysis was also shared in the Supplemental Materials.
