# Supplementary material for: Effect of Spirulina Meal Supplementation on Growth Performance and Feed Utilization in Fish and Shrimp: A Meta-Analysis
Source: Aquac Nutr. 2022 Nov 3;2022:8517733. doi: 10.1155/2022/8517733 (PMC9973199; doi:10.1155/2022/8517733)
Supplement: Supplementary Materials — The detailed information of included studies in this meta-analysis was listed in Table S1 of Supplemental Materials. The meta-analysis for CF and HSI were presented in Table S2 and Figure S7. In addition, publication bias for each outcome indicator (FBW, SGR, FCR, PER, CF, and HSI) in this study evaluated by methods of Egger's regression test, Begg's rank correlation test, and funnel plot were shown in Tables S3-S8 and Figures S1-S6, respectively. Furthermore, R code in this meta-analysis was also shared in the Supplemental Materials. [file 8517733.f1.docx]

Table S1. Summary of details of the studies included in this meta-analysis

| Species | Trophic level | Primary response criteria | Replicates | Habitat | Trial duration  (d) | IBW  (g) | FM content in control diet (%) | FM content in experimental diet (%) | Spirulina addition level (%) | References |
| --- | --- | --- | --- | --- | --- | --- | --- | --- | --- | --- |
| *Amphilophus citrinellus* × *Cichlasoma trimaculatum* | 3.2 | FBW, SGR | 3 | Freshwater | 90 | 12.09 | 32.00 | 26.50, 25.00 | 6.00, 12.00 | (Sornsupharp et al., 2015) |
| *Astronotus ocellatus* | 2.8 | FBW, FCR, SGR, PER | 3 | Freshwater | 56 | 10.41 | 55.00 | 53.63, 52.25, 49.50 | 1.38, 2.75, 5.50 | (Mohammadiazarm et al., 2021) |
| *Barilius bendelisis* | 3.4 | FBW, FCR, SGR | 3 | Freshwater | 60 | 5.63 | 20.27 | 17.27, 15.27, 13.27, 10.27 | 3.00, 5.00, 7.00, 10.00 | (Jha et al., 2012) |
| *Carassius auratus* | 3 | FCR, SGR | 3 | Freshwater | 120 | 0.27 | 39.80 | 31.50 | 3.00 | (James et al., 2009) |
| *Carassius auratus gibelio* | 2.5 | FBW, FCR, SGR | 3 | Freshwater | 46 | 15.37 | 12.00 | 9.00, 6.00, 0.00 | 3.38, 6.76, 13.52 | (Cao et al., 2018) |
|  | 2.5 | FBW, FCR, SGR, PER, CF | 3 | Freshwater | 60 | 5.00 | 20.00 | 10.00, 5.00, 0.00 | 9.87, 14.82, 19.76 | (Cao et al., 2018) |
|  | 2.5 | FBW, FCR, SGR, CF | 3 | Freshwater | 60 | 3.22 | 50.31 | 40.25, 30.18, 20.12, 10.06, 0 | 11.94, 23.88, 35.83, 47.76, 59.70 | (Cao et al., 2016) |
| *Clarias gariepinus* | 3.8 | FBW, FCR, SGR, PER, HSI, CF | 3 | Freshwater | 84 | 42.07 | 25.00 | 12.50, 6.25 | 12.50, 18.75 | (Raji et al., 2018) |
|  | 3.8 | FBW, FCR, SGR, PER | 3 | Freshwater | 42 | 58.00 | 30.00 | 0.00 | 30.00 | (Raji et al., 2020) |
| *Clarias macrocephalus* | 3.7 | FBW, FCR, SGR | 3 | Freshwater | 120 | 19.00 | 32.00 | 27.00, 22.00 | 5.00, 10.00 | (Chainapong et al., 2018) |
| *Cyprinus carpio* | 3.1 | FBW, FCR, SGR, PER | 3 | Freshwater | 56 | 30.00 | 15.00 | 12.50, 10.00, 7.50, 5.00 | 2.50, 5.00, 7.50, 10.00 | (Ansarifard et al., 2018) |
|  | 3.1 | FBW, FCR, SGR, PER | 3 | Freshwater | 45 | 4.21 | 35.00 | 35.00, 35.00, 35.00 | 1.00, 2.00, 3.00 | (Ramakrishnan et al., 2008) |

Table S1. Continued

| Species | Trophic level | Primary response criteria | Replicates | Habitat | Trial duration  (d) | IBW  (g) | FM content in control diet (%) | FM content in experimental diet (%) | Spirulina addition level (%) | References |
| --- | --- | --- | --- | --- | --- | --- | --- | --- | --- | --- |
| *Cyprinus carpio* | 3.1 | FBW, FCR, SGR, HSI | 3 | Freshwater | 42 | 25.00 | 37.00 | 37.00 | 0.08 | (Ren et al., 2021) |
|  | 3.1 | FCR, SGR, HSI, CF | 3 | Freshwater | 99 | 18.04 | 15.00 | 15.00 | 7.50 | (Sun et al., 2012) |
| *Cyrtocara moorii* | 3.4 | FBW, FCR, SGR, PER, CF | 3 | Freshwater | 84 | 3.15 | 55.00 | 52.0, 49.0, 46.0, 43.0 | 3.00, 6.00, 9.00, 12.00 | (Erdogan 2019) |
| *Dicentrarchus labrax* | 3.5 | FBW, FCR, SGR, PER | 3 | Marine | 60 | 5.74 | 35.50 | 35.50, 35.50, 35.50 | 1.00, 2.50, 5.00 | (Guroy et al., 2022) |
| *Fenneropenaeus chinensis* | 2.46 | FBW | 3 | Marine | 35 | 8.00 | NA | NA | 3.00 | (Kim et al., 2006) |
| *Litopenaeus vannamei* | 2.6 | FBW, FCR, SGR | 3 | Marine | 28 | 3.81 | NA | NA | 1.00, 1.00, 1.00 | (Liu et al., 2022) |
|  | 2.6 | FBW, FCR, SGR, PER | 3 | Marine | 50 | 0.70 | 40.0 | 30.0, 20.0, 10.0, 0.0 | 10.0, 20.0, 30.0, 40.0 | (Macias-Sancho et al., 2014) |
|  | 2.6 | FBW, FCR, PER | 4 | Marine | 72 | 3.89 | 13.87, 9.24 | 13.87, 9.24 | 0.50, 0.50 | (Silva-Neto et al., 2012) |
|  | 2.6 | FCR, SGR | 3 | Marine | 56 | 2.60 | 40.00 | 30.0, 20.0, 10.0, 0.0 | 8.0, 15.5, 23.0, 30.0 | (Pakravan et al., 2017) |
| *Marobrachium rosenbergii* | 3.38 | FBW, FCR, SGR, PER, HSI, CF | 3 | Freshwater | 60 | 0.33 | 35.00 | 28.0, 21.0, 14.0, 7.0 | 7.13, 14.27, 21.40, 28.54 | (Qu et al., 2021) |
| *Marsupenaeus japonicus* | 3.38 | FBW | 3 | Marine | 63 | 0.40 | 50.00 | 50.00 | 0.94, 1.88 | (Chien and Shiau 2005) |
| *Megalobrama amblycephala* | 3.3 | FBW, FCR, PER, HSI, CF | 3 | Freshwater | 65 | 21.60 | 8.00 | 7.34, 6.66, 5.34, 2.67, 0.00 | 0.75, 1.50, 3.00, 6.00, 9.00 | (Jiang et al., 2022) |
| *Mugil liza* | 2.0 | FBW, FCR, SGR, PER | 3 | Marine | 80 | 0.26 | 39.00 | 27.0, 19.5, 12.0, 0.0 | 12.0, 19.5, 27.0, 39.0 | (Rosas et al., 2019) |
|  | 2.0 | FBW, FCR, SGR, PER | 3 | Marine | 75 | 0.48 | 45.00 | 42.75, 40.50 | 2.25, 4.50 | (Rosas et al., 2019) |
| *Neocaridina davidi* | 1.56 | FBW, FCR, SGR, CF | 3 | Freshwater | 330 | 0.45mg | 31.00 | 30.0, 28.0, 26.0, 23.0, 21.0 | 1.0, 3.0, 5.0, 8.0, 10.0 | (Namaei Kohal et al., 2018) |
| *Oncorhynchus mykiss* | 4.1 | FBW, FCR, SGR | 3 | Freshwater | 84 | 135.68 | 38.00, 22.00 | 36.00, 20.00 | 4.00, 4.00 | (Güroy et al., 2019) |

Table S1. Continued

| Species | Trophic level | Primary response criteria | Replicates | Habitat | Trial duration  (d) | IBW  (g) | FM content in control diet (%) | FM content in experimental diet (%) | Spirulina addition level (%) | References |
| --- | --- | --- | --- | --- | --- | --- | --- | --- | --- | --- |
| *Oncorhynchus mykiss* | 4.1 | FBW, FCR, SGR, CF | 3 | Freshwater | 56 | 12.60 | NA | NA | 0.025, 0.05, 0.10, 0.25 | (Kermani et al., 2020) |
|  | 4.1 | FBW, CF | 3 | Freshwater | 49 | 17.18 | 45.00 | 42.50, 40.00 | 2.50, 5.00 | (Sheikhzadeh et al., 2019) |
| *Oncorhynchus mykiss* | 4.1 | FBW, FCR, SGR | 3 | Freshwater | 70 | 101 | 42.00 | 39.5, 37.0, 34.5, 32.0 | 2.5, 5.0, 7.5, 10.0 | (Teimouri et al., 2013) |
|  | 4.1 | FBW, SGR | 4 | Freshwater | 84 | 12.4 | 46.50 | 23.20 | 23.30 | (Twibell et al., 2020) |
| *Oplegnathus fasciatus* | 3.6 | FBW, FCR, SGR, PER, HSI, CF | 3 | Marine | 56 | 57.0 | 53.00 | 46.00, 39.00, 33.00 | 9.00, 18.00, 26.00 | (Kim et al., 2013) |
| *Oreochromis niloticus* | 2.0 | FBW, FCR, SGR, PER | 3 | Freshwater | 84 | 1.88 | 9.10 | 9.10 | 0.13, 0.25, 0.50, 0.75, 1.00 | (Abdel-Tawwab and Ahmad 2009) |
|  | 2.0 | FBW, FCR, SGR | 3 | Freshwater | 56 | 20.20 | 16.00 | 16.00 | 2.00, 4.00 | (Abdel-Tawwab et al., 2021) |
|  | 2.0 | FBW, FCR, SGR, PER | 2 | Freshwater | 84 | 15.98 | 32.00 | 28.00, 24.00, 21.00 | 4.00, 8.00, 12.00 | (Abdel-Warith and Elsayed 2019) |
|  | 2.0 | FBW, FCR, SGR | 3 | Freshwater | 60 | 24.55 | NA | NA | 0.10 | (Al-Deriny et al., 2020) |
|  | 2.0 | FBW, FCR, SGR | 3 | Freshwater | 60 | 24.95 | 2.32 | 2.32 | 0.10 | (Al-Deriny et al., 2020) |
|  | 2.0 | FBW, FCR, SGR, PER | 3 | Freshwater | 84 | 7.08 | 15.00 | 15.00 | 1.00 | (Belal et al., 2012) |
|  | 2.0 | FBW, SGR | 3 | Freshwater | 76 | 26.84 | 0.00 | 0.00 | 0.25, 0.50 | (Grassi et al., 2018) |
|  | 2.0 | FBW, FCR, PER | 3 | Freshwater | 70 | 20.03 | 18.00 | 18.00 | 0.01, 0.1, 1.0, 1.0 | (Mabrouk et al., 2022) |

Table S1. Continued

| Species | Trophic level | Primary response criteria | Replicates | Habitat | Trial duration  (d) | IBW  (g) | FM content in control diet (%) | FM content in experimental diet (%) | Spirulina addition level (%) | References |
| --- | --- | --- | --- | --- | --- | --- | --- | --- | --- | --- |
| *Oreochromis niloticus* | 2.0 | FBW, FCR, SGR, PER, HSI | 3 | Freshwater | 83 | 9.30 | 20.00 | 20.00 | 1.00, 2.00 | (Mahmoud et al., 2018) |
|  | 2.0 | FBW, FCR | 2 | Freshwater | 50 | 2.63 mg | NA | NA | 1.00 | (Plaza et al., 2018) |
|  | 2.0 | FBW | 2 | Freshwater | 50 | 109.1 | 35.00 | 35.00 | 3.00 | (Plaza et al., 2019) |
|  | 2.0 | FBW, FCR, SGR, PER, HSI, CF | 3 | Freshwater | 60 | 12.50 | 6.00 | 6.00 | 0.10 | (Shalata et al., 2021) |
| *Oreochromis niloticus* | 2.0 | FBW, SGR | 4 | Freshwater | 102 | 3.90 | NA | NA | 0.40, 0.80, 1.20 | (Siringi et al., 2021) |
|  | 2.0 | FBW, FCR, SGR, HSI, CF | 3 | Freshwater | 60 | 0.89 | 56.50 | 39.5, 30.0, 14.0, 0.0 | 19.0, 30.0, 39.0, 48.0 | (Velasquez et al., 2016) |
|  | 2.0 | FBW, FCR, SGR, PER | 3 | Freshwater | 65 | 0.23 | 25.00 | 12.50, 6.50, 0.00 | 14.0, 22.5, 28.0 | (El-Sheekh et al., 2014) |
| *Pagrus pagrus* | 3.9 | FBW, HSI | 2 | Marine | 84 | 131.9 | 62.70 | 58.60 | 5.00 | (Chatzifotis et al., 2011) |
| *Pangasinodon gigas* | 2.3 | FCR | 3 | Freshwater | 240 | 6000 | 16.00 | 7.00 | 9.00 | (Meng-umphan 2008) |
| *Pelteobagrus fulvidraco* | 3.5 | FBW, FCR, SGR, HSI, CF | 3 | Freshwater | 50 | 3.10 | 30.0 | 24.0, 18.0, 12.0, 6.0, 0.0 | 5.76, 11.51, 17.27, 23.03, 28.79 | (Liu et al., 2019) |
|  | 3.5 | FBW, FCR, SGR | 3 | Freshwater | 56 | 20.87 | 20.00 | 20.00 | 0.5, 1.0, 2.0, 4.0 | (Liu et al., 2020) |
|  | 3.5 | FBW, FCR, SGR | 3 | Freshwater | 56 | 20.87 | 20.00 | 20.00 | 4.00, 4.00, 0.40 | (Liu et al., 2021) |
|  | 3.5 | FBW, FCR, SGR | 3 | Freshwater | 35 | 4.79 | 30.00 | 28.24 | 2.00 | (Liu et al., 2021) |
|  | 3.5 | FBW, FCR, SGR, HSI, CF | 3 | Freshwater | 56 | 34.84 | 18.40 | 18.13 | 1.00 | (Xia et al., 2021) |

Table S1. Continued

| Species | Trophic level | Primary response criteria | Replicates | Habitat | Trial duration  (d) | IBW  (g) | FM content in control diet (%) | FM content in experimental diet (%) | Spirulina addition level (%) | References |
| --- | --- | --- | --- | --- | --- | --- | --- | --- | --- | --- |
| *Penaeus monodon* | 2.75 | FBW, SGR | 2 | Freshwater | 84 | 1.24 | 48.50 | 48.50 | 0.05, 0.10 | (Abdel-Warith et al., 2020) |
|  | 2.75 | FBW, FCR, SGR | 3 | Marine | 30 | 0.20 | 37.00 | 37.00 | 1.00 | (Kolanchinathan et al., 2022) |
|  | 2.75 | FCR, SGR, PER | 3 | Marine | 60 | NA | 35.00 | 30.0, 25.0, 20.0, 15.0 | 5.0, 10.0, 15.0, 20.0 | (Sivakumar et al., 2018) |
| *Piaractus mesopotamicus* | 2 | FBW, FCR, SGR, PER, HSI | 3 | Freshwater | 45 | 0.97 | 6.00 | 4.00, 2.00, 0.00 | 2.00, 4.00, 6.00 | (Carneiro et al., 2022) |
| *Salmo trutta caspius* | 3.5 | FCR, SGR, PER, CF | 3 | Freshwater | 70 | 11.00 | 66.00 | 64.68, 63.36, 62.04, 60.72 | 1.32, 2.64, 3.96, 5.28 | (Roohani et al., 2019) |
|  | 3.5 | FCR, SGR, CF | 3 | Marine | 70 | 11.00 | 65.89 | 64.57, 63.24, 61.94, 60.62 | 1.32, 2.65, 3.95, 5.27 | (Roohani et al., 2020) |
| *Solea solea* | 3.2 | FBW, SGR, CF | 3 | Marine | 28 | 9.94 mg | 65.00 | 65.00 | 5.00 | (Shawky et al., 2021) |
|  | 3.7 | FBW, FCR, SGR, PER, CF | 3 | Marine | 128 | 20.51 | 37.42 | 35.84, 34.06 | 2.00, 4.00 | (Galafat et al., 2020) |
| *Trichopodus trichopterus* | 2.7 | FBW, FCR, SGR, CF | 3 | Freshwater | 102 | 1.29 | 36.00 | 33.5, 31.0, 26.0, 16.0 | 2.5, 5.0, 10.0, 20.0 | (Khanzadeh et al., 2016) |

**References**

Abdel-Tawwab M, Ahmad MH. Live *Spirulina* (*Arthrospira platensis*) as a growth and immunity promoter for Nile tilapia, *Oreochromis niloticus* (L.), challenged with pathogenic *Aeromonas hydrophila*. Aquaculture Research 2009;40:1037-1046.

Abdel-Tawwab M, El-Saadawy HA, El-Belbasi HI, Abd El-Hameed SAA, Attia AA. Dietary spirulina (*Arthrospira platenesis*) mitigated the adverse effects of imidacloprid insecticide on the growth performance, haemato-biochemical, antioxidant, and immune responses of Nile tilapia. Comparative Biochemistry and Physiology Part C: Toxicology & Pharmacology 2021;247:109067.

Abdel-Warith A-WA, Fath El-Bab AF, Younis E-SMI, Al-Asgah NA, Allam HY, Abd-Elghany MF, Shata YHM, Shamlol FS. Using of chitosan nanoparticles (CsNPs), Spirulina as a feed additives under intensive culture system for black tiger shrimp (*Penaeus monodon*). Journal of King Saud University - Science 2020;32:3359-3363.

Abdel-Warith A, Elsayed E. Use of *Arthrospira platensis* as a feed additive to improve growth performance, feed utilization, body composition, and immune response of Nile Tilapia, *Oreochromis niloticus*. 2019.

Al-Deriny SH, Dawood MAO, Elbialy ZI, El-Tras WF, Mohamed RA. Selenium nanoparticles and spirulina alleviate growth performance, hemato-biochemical, immune-related genes, and heat shock protein in Nile Tilapia (*Oreochromis niloticus*). Biological Trace Element Research 2020;198:661-668.

Al-Deriny SH, Dawood MAO, Zaid AAA, El-Tras WF, Paray BA, Van Doan H, Mohamed RA. The synergistic effects of *Spirulina platensis* and *Bacillus amyloliquefaciens* on the growth performance, intestinal histomorphology, and immune response of Nile tilapia (*Oreochromis niloticus*). Aquaculture Reports 2020;17:100390.

Ansarifard F, Rajabi Islami H, Shamsaie Mehrjan M. Effects of Arthrospira platensis on growth, skin color and digestive enzymes of Koi, *Cyprinus carpio*. Iranian Journal of Fisheries Sciences 2018;17:381-393.

Belal EB, El-Hais AMA. Use of spirulina (*Arthrospira fusiformis*) for promoting growth of Nile Tilapia fingerlings. African Journal of Microbiology Research 2012;6:6423-6431.

Cao S, Han D, Xie S, Jin J, Liu H, Yang Y, Zhu X. Effects of dietary fishmeal replacement with spirulina platensis powder on the growth performance, feed utilization and protein deposition in juvenile gibel carp (*Carassis auratus gibelio* var. CAS). Acta Hydrobiologica Sinica 2016;40(4):647-654.

Cao SP, Zou T, Zhang PY, Han D, Jin JY, Liu HK, Yang YX, Zhu XM, Xie SQ. Effects of dietary fishmeal replacement with *Spirulina platensis* on the growth, feed utilization, digestion and physiological parameters in juvenile gibel carp (*Carassis auratus gibelio* var. CAS III). Aquaculture Research 2018;49:1320-1328.

Cao S, Zhang P, Zou T, Fei S, Han D, Jin J, Liu H, Yang Y, Zhu X, Xie S. Replacement of fishmeal by spirulina *Arthrospira platensis* affects growth, immune related-gene expression in gibel carp (*Carassius auratus gibelio* var. CAS III), and its challenge against *Aeromonas hydrophila* infection. Fish & Shellfish Immunology 2018;79:265-273.

Carneiro WF, Castro TFD, Reichel T, de Castro Uzeda PL, Martínez-Palacios CA, Murgas LDS. Diets containing *Arthrospira platensis* increase growth, modulate lipid metabolism, and reduce oxidative stress in pacu (*Piaractus mesopotamicus*) exposed to ammonia. Aquaculture 2022;547:737402.

Chainapong T, Traichaiyaporn S, Deming RL. Effect of Dietary *Spirulina platensis* on the fatty acid composition in flesh and ovary of walking catfish (*Clarias macrocephalus*). Chiang Mai Journal of Science 2018;45:129-135.

Chatzifotis S, Vaz Juan I, Kyriazi P, Divanach P, Pavlidis M. Dietary carotenoids and skin melanin content influence the coloration of farmed red porgy (*Pagrus pagrus*). Aquaculture Nutrition 2011;17:e90-e100.

Chien YH, Shiau WC. The effects of dietary supplementation of algae and synthetic astaxanthin on body astaxanthin, survival, growth, and low dissolved oxygen stress resistance of kuruma prawn, *Marsupenaeus japonicus* Bate. Journal of Experimental Marine Biology and Ecology 2005;318:201-211.

El-Sheekh M, E-Shourbagy I, Shalaby S, Hosny S. Effect of feeding *Arthrospira platensis* (spirulina) on growth and carcass composition of hybrid red tilapia (*Oreochromis niloticus* × *Oreochromis mossambicus*). Turkish Journal of Fisheries and Aquatic Sciences 2014;14:471-478.

Erdogan F. Effects of *Spirulina platensis* as a feed additive on growth and coloration of blue dolphin cichlids (*Cyrtocara moorii* Boulunger, 1902). Aquaculture Research 2019;50:2326-2332.

Galafat A, Vizcaino AJ, Saez MI, Martinez TF, Jerez-Cepa I, Mancera JM, Alarcon FJ. Evaluation of*Arthrospira* sp. enzyme hydrolysate as dietary additive in gilthead seabream (*Sparus aurata*) juveniles. Journal of Applied Phycology 2020;32:3089-3100.

Grassi TLM, Oliveira DL, Paiva NM, Diniz JCP, Bosco AM, Pereira AAF, Menezes ARP, Valadares TC, Pastor RCP, Ciarlini PC, Gonçalves GS, Villarroel M, Ponsano EHG. Microbial biomass as an antioxidant for tilapia feed. Aquaculture Research 2018;49:2881-2890.

Guroy B, Guroy D, Bilen S, Kenanoglu ON, Sahin I, Terzi E, Karadal OMantoglu S. Effect of dietary Spirulina (*Arthrospira platensis*) on the growth performance, immune-related gene expression and resistance to *Vibrio anguillarum* in European seabass (*Dicentrarchus labrax*). Aquaculture Research 2022;53(6):2263-2274.

Güroy B, Güroy D, Mantoğlu S, Çelebi K, Şahin OI, Kayalı S, Canan B. Dietary *Spirulina* (*Arthrospira platensis*, Gomont, 1892) improved the flesh quality and shelf life of rainbow trout (*Oncorhynchus mykiss*, Walbaum, 1792) fed fish meal or plant-based diet. Aquaculture Research 2019;50:2519-2527.

James R, Vasudhevan I, Sampath K. Interaction of *Spirulina* with different levels of vitamin E on growth, reproduction, and coloration in goldfish (*Carassius auratus*). 2009; 61:330-338.

Jha GN, Sarma D, Qureshi TA. Effect of spirulina (*Spirulina platensis*) and marigold (*Tagetes erecta*) fortified diets on growth, body composition and total carotenoid content of *Barilius bendelisis*. Indian Journal of Animal Sciences 2012;82:336-340.

Jiang W, Miao L, Lin Y, Ci L, Liu B, Ge X. *Spirulina* (*Arthrospira*) *platensis* as a protein source could improve growth, feed utilisation and digestion and physiological status in juvenile blunt snout bream (*Megalobrama amblycephala*). Aquaculture Reports 2022;22:100932.

Kermani P, Babaei S, Abedian-Kenari A, Hedayati M. Growth performance, plasma parameters and liver antioxidant enzymes activities of Rainbow trout (*Oncorhynchus mykiss*) juvenile fed on *Spirulina platensis* extract. Iranian Journal Of Fisheries Sciences 2020;19:1463-1478.

Khanzadeh M, Fereidouni AE, Berenjestanaki SS. Effects of partial replacement of fish meal with *Spirulina platensis* meal in practical diets on growth, survival, body composition, and reproductive performance of three-spot gourami (*Trichopodus trichopterus*) (Pallas, 1770). Aquaculture International 2016;24:69-84.

Kim CJ, Yoon SK, Kim HI, Park YH, Oh HM. Effect of Spirulina platensis and probiotics as feed additives on growth of shrimp Fenneropenaeus chinensis. Journal of Microbiology and Biotechnology 2006;16:1248-1254.

Kim SS, Rahimnejad S, Kim KW, Lee KJ. Partial replacement of fish meal with spirulina pacifica in diets for parrot fish (*Oplegnathus fasciatus*). Turkish Journal of Fisheries and Aquatic Sciences 2013;13:197-204.

Kolanchinathan P, Kumari PR, Raja K, John G, Balasundaram A. Analysis of feed composition and growth parameters of *Penaeus monodon* supplemented with two probiotic species and formulated diet. Aquaculture 2022;549:737740.

Liu C, Li Y, Chen Z, Yuan L, Liu H, Han D, Jin J, Yang Y, Hu Q, Zhu X, Xie S. Effects of dietary whole and defatted *Arthrospira platensis* (*Cyanobacterium*) on growth, body composition and pigmentation of the yellow catfish *Pelteobagrus fulvidraco*. Journal of Applied Phycology 2021;33:2251-2259.

Liu C, Liu H, Han D, Xie S, Jin J, Yang Y, Zhu X. Effects of dietary Arthrospira platensis supplementation on the growth performance, antioxidation and immune related-gene expression in yellow catfish (*Pelteobagrus fulvidraco*). Aquaculture Reports 2020;17:100297.

Liu C, Liu H, Xu W, Han D, Xie S, Jin J, Yang Y, Zhu X. Effects of dietary Arthrospira platensis supplementation on the growth, pigmentation, and antioxidation in yellow catfish (*Pelteobagrus fulvidraco*). Aquaculture 2019;510:267-275.

Liu C, Liu HK, Zhu XM, Han D, Jin JY, Yang YX, Xie SQ. the effects of *Arthrospira platensis* and lutein on the growth, antioxidant capacity and pigmentation in hybrid yellow catfish (*Pelteobagrus fulvidraco*♀ × *Pelteobaggrus vachelli*♂). Acta Hydrobiologica Sinica 2021;45(5):1024-1033.

Liu L, Cai X, Ai Y, Li J, Long H, Ren W, Huang A, Zhang X, Xie ZY. Effects of *Lactobacillus pentosus* combined with *Arthrospira platensis* on the growth performance, immune response, and intestinal microbiota of *Litopenaeus vannamei*. Fish & Shellfish Immunology 2022;120:345-352.

Mabrouk MM, Ashour M, Labena A, Zaki MAA, Abdelhamid AF, Gewaily MS, Dawood MAO, Abualnaja KM, Ayoub HF. Nanoparticles of *Arthrospira platensis* improves growth, antioxidative and immunological responses of Nile tilapia (*Oreochromis niloticus*) and its resistance to *Aeromonas hydrophila*. Aquaculture Research 2022;53:125-135.

Macias-Sancho J, Poersch LH, Bauer W, Romano LA, Wasielesky W, Tesser MB. Fishmeal substitution with Arthrospira (*Spirulina platensis*) in a practical diet for *Litopenaeus vannamei*: Effects on growth and immunological parameters. Aquaculture 2014;426-427:120-125.

Mahmoud MMA, El-Lamie MMM, Kilany OE, Dessouki AA. Spirulina (*Arthrospira platensis*) supplementation improves growth performance, feed utilization, immune response, and relieves oxidative stress in Nile tilapia (*Oreochromis niloticus*) challenged with *Pseudomonas fluorescens*. Fish & Shellfish Immunology 2018;72:291-300.

Meng-umphan K. Production of Generation-2 Mekong giant catfish (Pangasinodon gigas) cultured with Spirulina sp. 2008.

Mohammadiazarm H, Maniat M, Ghorbanijezeh K, Ghotbeddin N. Effects of spirulina powder (*Spirulina platensis*) as a dietary additive on Oscar fish, *Astronotus ocellatus*: assessing growth performance, body composition, digestive enzyme activity, immune-biochemical parameters, blood indices and total pigmentation. Aquaculture Nutrition 2021;27:252-260.

Namaei Kohal M, Esmaeili Fereidouni A, Firouzbakhsh F, Hayati I. Effects of dietary incorporation of *Arthrospira* (*Spirulina*) *platensis* meal on growth, survival, body composition, and reproductive performance of red cherry shrimp *Neocaridina davidi* (Crustacea, Atyidae) over successive spawnings. Journal of Applied Phycology 2018;30:431-443.

Pakravan S, Akbarzadeh A, Sajjadi MM, Hajimoradloo A, Noori F. Partial and total replacement of fish meal by marine microalga *Spirulina platensis* in the diet of Pacific white shrimp *Litopenaeus vannamei*: Growth, digestive enzyme activities, fatty acid composition and responses to ammonia and hypoxia stress. Aquaculture Research 2017;48:5576-5586.

Plaza I, García JL, Galán B, de la Fuente J, Bermejo-Poza R, Villarroel M. Effect of Arthrospira supplementation on *Oreochromis niloticus* gut microbiota and flesh quality. Aquaculture Research 2019;50:1448-1458.

Plaza I, García JL, Villarroel M. Effect of spirulina (*Arthrospira platensis*) supplementation on tilapia (*Oreochromis niloticus*) growth and stress responsiveness under hypoxia. Spanish Journal of Agricultural Research 2018;16(1):e0606-e0606.

Qu YH, Li X, Wang HL, Rong JF, Lei Y, Li DD, Zhang ED, Wu GQ. Effects of partial replacement of fish meal by Spirulina on growth performance, nutrient apparent digestibility coefficients, whole-body composition and serum biochemical indices of *Macrobrachium rosenbergii*. Chinese Journal of Animal Nutrition 2021;33(4):2187-2198.

Raji AA, Alaba PA, Yusuf H, Abu Bakar NH, Mohd Taufek N, Muin H, Alias Z, Milow P, Abdul Razak S. Fishmeal replacement with *Spirulina Platensis* and *Chlorella vulgaris* in African catfish (*Clarias gariepinus*) diet: Effect on antioxidant enzyme activities and haematological parameters. Research in Veterinary Science 2018;119:67-75.

Raji AA, Jimoh WA, Abu Bakar NH, Taufek NHM, Muin H, Alias Z, Milow P, Razak SA. Dietary use of Spirulina (*Arthrospira*) and *Chlorella* instead of fish meal on growth and digestibility of nutrients, amino acids and fatty acids by African catfish. Journal of Applied Phycology 2020;32:1763-1770.

Ramakrishnan CM, Haniffa M, Manohar M, Dhanaraj M, Arockiaraj AJ, Arunsingh S. Effects of probiotics and spirulina on survival and growth of juvenile common carp (*Cyprinus carpio*). 2008.

Ren Ht, Zhao Xj, Huang Y, Xiong JL. Combined effect of *Spirulina* and ferrous fumarate on growth parameters, pigmentation, digestive enzyme activity, antioxidant enzyme activity and fatty acids composition of Yellow River carp (*Cyprinus carpio*). Aquaculture Reports 2021;21:100776.

Roohani AM, Abedian Kenari A, Fallahi Kapoorchali M, Borani MS, Zoriezahra SJ, Smiley AH, Esmaeili M, Rombenso AN. Effect of spirulina *Spirulina platensis* as a complementary ingredient to reduce dietary fish meal on the growth performance, whole-body composition, fatty acid and amino acid profiles, and pigmentation of Caspian brown trout (*Salmo trutta caspius*) juveniles. Aquaculture Nutrition 2019;25:633-645.

Roohani AM, Kapoorchali MF, Kenari AA, Borani MS, Zorriezahra MJ. Hematite-biochemical and immune response of Caspian brown trout (*Salmo troutta caspius*, Kessler, 1877) juveniles fed different levels of spirulina (*Spirulina platensis*). Iranian Journal of Fisheries Sciences 2020;19:1153-1174.

Rosas VT, Bessonart M, Romano LA, Tesser TB. Fishmeal substitution for *Arthrospira platensis* in juvenile mullet (*Mugil liza*) and its effects on growth and non-specific immune parameters. Rev Colomb Cienc Pec 2019;32:3-13.

Rosas VT, Monserrat JM, Bessonart M, Magnone L, Romano LA, Tesser MB. Comparison of β-carotene and Spirulina (*Arthrospira platensis*) in mullet (*Mugil liza*) diets and effects on antioxidant performance and fillet colouration. Journal of Applied Phycology 2019;31:2391-2399.

Shalata HA, Bahattab O, Zayed MM, Farrag F, Salah AS, Al-Awthan YS, Ebied NA, Mohamed RA. Synergistic effects of dietary sodium butyrate and *Spirulina platensis* on growth performance, carcass composition, blood health, and intestinal histomorphology of Nile tilapia (*Oreochromis niloticus*). Aquaculture Reports 2021;19:100637.

Shawky WA, El-Sayed HS, Saleh NE, Ismael A, AEl-Sayed A-FM. Evaluation of microalgae-supplemented diets and enriched decapsulated artemia cyst powder as novel diets for post-weaned common sole (*Solea solea*) larvae. Aquaculture Nutrition 2021;27:1042-1051.

Sheikhzadeh N, Mousavi S, Khani Oushani A, Firouzamandi M, Mardani K. Spirulina platensis in rainbow trout (*Oncorhynchus mykiss*) feed: effects on growth, fillet composition, and tissue antioxidant mechanisms. Aquaculture International 2019;27:1613-1623.

Silva-Neto JF, Nunes AJP, Sabry-Neto H, Sá MVC. Spirulina meal has acted as a strong feeding attractant for Litopenaeus vannamei at a very low dietary inclusion level. Aquaculture Research 2012;43:430-437.

Siringi JO, Turoop L, Njonge F. Growth and biochemical response of Nile tilapia (*Oreochromis niloticus*) to spirulina (*Arthrospira platensis*) enhanced aquaponic system. Aquaculture 2021;544:737134.

Sivakumar N, Sundararaman M, Selvakumar G. Evaluation of growth performance of Penaeus monodon (Fabricius) fed diet with partial replacement of fishmeal by Spirulina platensis (Sp) meal. Indian Journal of Animal Research 2018;52:1721-1726.

Sornsupharp B, Lomthaisong K, Dahms HU, Sanoamuang LO. Effects of dried fairy shrimp Streptocephalus sirindhornae meal on pigmentation and carotenoid deposition in flowerhorn cichlid; *Amphilophus citrinellus* (Günther, 1864) × *Cichlasoma trimaculatum* (Günther, 1867). Aquaculture Research 2015;46:173-184.

Sun X, Chang Y, Ye Y, Ma Z, Liang Y, Li T, Jiang N, Xing W, Luo L. The effect of dietary pigments on the coloration of Japanese ornamental carp (koi, *Cyprinus carpio* L.). Aquaculture 2012;342-343:62-68.

Teimouri M, Amirkolaie AK, Yeganeh S. The effects of *Spirulina platensis* meal as a feed supplement on growth performance and pigmentation of rainbow trout (*Oncorhynchus mykiss*). Aquaculture 2013;396-399:14-19.

Twibell R, Johnson R, Hyde N, Gannam A. Evaluation of *Spirulina* and plant oil in diets for juvenile steelhead (*Oncorhynchus mykiss*). Aquaculture 2020;528:735598.

Velasquez SF, Chan MA, Abisado RG, Traifalgar RFM, Tayamen MM, Maliwat GCF, Ragaza JA. Dietary Spirulina (*Arthrospira platensis*) replacement enhances performance of juvenile Nile tilapia (*Oreochromis niloticus*). Journal of Applied Phycology 2016;28:1023-1030.

Xia Y, Liu C, Fei S, Liu H, Han D, Jin J, Yang Y, Zhu X, Xie S. *Arthrospira platensis* additive enhances the growth performance and antioxidant response in hybrid yellow catfish (*Pelteobagrus fulvidraco*♀ × *Pelteobagrus vachelli*♂). Aquaculture Reports 2021;20:100721.

**Table S2**. Effect size calculation for CF and HSI comparison through random-effect model

|  | Effect size (random-effect model) for CF | | | | | | Effect size (random-effect model) for HSI | | | | | | |  |
| --- | --- | --- | --- | --- | --- | --- | --- | --- | --- | --- | --- | --- | --- | --- |
|  | *k* | *I*^2^ | Hedges’ *g* value | SE | C.L. | *P*-value | | *k* | *I*^2^ | Hedges’ *g* value | SE | C.L. | *P*-value |  |
| All species | 65 | 54.52 | -0.030 | 0.073 | -0.173 to 0.114 | 0.688 | | 34 | 71.58 | -0.032 | 0.168 | -0.360 to 0.297 | 0.849 |  |
| ***Subgroups*** |  |  |  |  |  |  | |  |  |  |  |  |  |  |
| *Species category* |  |  |  |  |  |  | |  |  |  |  |  |  |  |
| Fish species | 56 | 49.31 | -0.110 | 0.070 | -0.248 to 0.027 | 0.116 | | 30 | 65.27 | 0.151 | 0.162 | -0.165 to 0.468 | 0.349 |  |
| Shrimps | 9 | 26.33 | 1.135 | 0.275 | 0.597 to 1.674 | < 0.0001 | | 4 | 0 | -1.244 | 0.263 | -1.760 to -0.729 | < 0.0001 |  |
| ***Fish + habitat*** |  |  |  |  |  |  | |  |  |  |  |  |  |  |
| **Freshwater fish** | 46 | 62.49 | -0.156 | 0.091 | -0.334 to 0.022 | 0.086 | | 26 | 66.21 | 0.155 | 0.176 | -0.190 to 0.499 | 0.379 |  |
| **Marine fish** | 10 | 0 | -0.023 | 0.105 | -0.229 to 0.182 | 0.823 | | 4 | 68.75 | 0.118 | 0.467 | -0.797 to 1.034 | 0.800 |  |
| *Supplemental types* |  |  |  |  |  |  | |  |  |  |  |  |  |  |
| Additives | 23 | 34.37 | -0.074 | 0.088 | -0.246 to 0.097 | 0.396 | | 11 | 21.65 | 0.117 | 0.185 | -0.245 to 0.479 | 0.526 |  |
| Ingredients | 42 | 60.79 | 0.005 | 0.108 | -0.207 to 0.216 | 0.964 | | 23 | 80.01 | -0.127 | 0.238 | -0.592 to 0.339 | 0.594 |  |
| *Trophic level* |  |  |  |  |  |  | |  |  |  |  |  |  |  |
| Low trophic level | 23 | 81.18 | -0.207 | 0.286 | -0.768 to 0.354 | 0.470 | | 11 | 42.74 | 0.048 | 0.261 | -0.464 to 0.560 | 0.854 |  |
| Medium trophic level | 36 | 35.54 | 0.086 | 0.075 | -0.061 to 0.233 | 0.252 | | 23 | 77.48 | -0.034 | 0.209 | -0.443 to 0.375 | 0.870 |  |
| High trophic level (*O. mykiss*) | 6 | 0 | 0.027 | 0.098 | -0.164 to 0.219 | 0.781 | |  |  |  |  |  |  |  |

*K,* sample size (no. of comparison); I^2^ percentage variation across studies due to heterogeneity; SE, standard error; C.L. confidence limits (lower and upper).

**Table S3:** Outcomes of Egger’s regression test and Begg’s rank correlation test to evaluate publication bias during the study for final body weight comparisons.

| **Final body weight** | Egger’s regression test | | Begge’s rank correlation test | |
| --- | --- | --- | --- | --- |
|  | z value | p-value | Kendall's tau | p-value |
| All species | 6.1297 | <0.0001 | 0.2365 | <0.0001 |
| Subgroups |  |  |  |  |
| ***Species category*** |  |  |  |  |
| Fish species | 2.3083 | 0.0210 | 0.2243 | 0.0002 |
| Shrimps | 8.1987 | <0.0001 | 0.2464 | 0.0968 |
| ***Fish + Habitat*** |  |  |  |  |
| Freshwater fish | 1.4809 | 0.1386 | 0.1686 | 0.0097 |
| Marine fish | 2.9084 | 0.0036 | 0.3000 | 0.1160 |
| ***Supplemental types*** |  |  |  |  |
| Additives | 6.0979 | <0.0001 | 0.2596 | 0.0008 |
| Ingredients | 3.2686 | 0.0011 | 0.2373 | 0.0035 |
| ***Trophic level*** |  |  |  |  |
| Low trophic level species | 4.8678 | <0.0001 | 0.2656 | 0.0005 |
| Medium trophic level species | 5.4659 | <0.0001 | 0.2615 | 0.0053 |
| High trophic level species | -2.5451 | 0.0109 | -0.1209 | 0.5906 |

**Table S4:** Outcomes of Egger’s regression test and Begg’s rank correlation test to evaluate publication bias during the study for specific growth rate comparisons.

| **Specific growth rate** | Egger’s regression test | | Begge’s rank correlation test | |
| --- | --- | --- | --- | --- |
|  | z value | p-value | Kendall's tau | p-value |
| All species | 3.3368 | 0.0008 | 0.1281 | 0.0205 |
| Subgroups |  |  |  |  |
| ***Species category*** |  |  |  |  |
| Fish species | 3.5987 | 0.0003 | 0.1268 | 0.0376 |
| Shrimps | -0.0061 | 0.9951 | 0.1323 | 0.3582 |
| ***Fish + Habitat*** |  |  |  |  |
| Freshwater fish | 2.6822 | 0.0073 | 0.0869 | 0.1826 |
| Marine fish | 4.1355 | <0.0001 | 0.4476 | 0.0208 |
| ***Supplemental types*** |  |  |  |  |
| Additives | 3.7594 | 0.0002 | 0.0751 | 0.3653 |
| Ingredients | 1.6643 | 0.0961 | 0.1543 | 0.0416 |
| ***Trophic level*** |  |  |  |  |
| Low trophic level species | 1.6986 | 0.0894 | 0.1698 | 0.0268 |
| Medium trophic level species | 4.7651 | <0.0001 | 0.1634 | 0.0701 |
| High trophic level species | -2.1624 | 0.0306 | -0.2290 | 0.3025 |

**Table S5:** Outcomes of Egger’s regression test and Begg’s rank correlation test to evaluate publication bias during the study for feed conversion ratio comparisons.

| **Feed conversion ratio** | Egger’s regression test | | Begge’s rank correlation test | |
| --- | --- | --- | --- | --- |
|  | z value | p-value | Kendall's tau | p-value |
| All species | -2.3354 | 0.0195 | -0.1494 | 0.0073 |
| Subgroups |  |  |  |  |
| ***Species category*** |  |  |  |  |
| Fish species | -1.9463 | 0.0516 | -0.1452 | 0.0182 |
| Shrimps | -2.3413 | 0.0192 | -0.2800 | 0.0465 |
| ***Fish + Habitat*** |  |  |  |  |
| Freshwater fish | -2.4111 | 0.0159 | -0.1233 | 0.0599 |
| Marine fish | 0.9494 | 0.3424 | -0.4945 | 0.0138 |
| ***Supplemental types*** |  |  |  |  |
| Additives | -2.4856 | 0.0129 | -0.1654 | 0.0431 |
| Ingredients | -1.5258 | 0.1271 | -0.1401 | 0.0720 |
| ***Trophic level*** |  |  |  |  |
| Low trophic level species | -0.1401 | 0.8886 | -0.1324 | 0.0802 |
| Medium trophic level species | -3.8185 | 0.0001 | -0.1165 | 0.2038 |
| High trophic level species | -3.9172 | <0.0001 | -0.7143 | 0.0084 |

**Table S6:** Outcomes of Egger’s regression test and Begg’s rank correlation test to evaluate publication bias during the study for protein efficiency ratio comparisons.

| **Protein efficiency ratio** | Egger’s regression test | | Begge’s rank correlation test | |
| --- | --- | --- | --- | --- |
|  | z value | p-value | Kendall's tau | p-value |
| All species | 5.1372 | <0.0001 | 0.2568 | 0.0010 |
| Subgroups |  |  |  |  |
| ***Species category*** |  |  |  |  |
| Fish species | 4.9536 | <0.0001 | 0.2910 | 0.0008 |
| Shrimps | -0.2647 | 0.7913 | -0.0769 | 0.7472 |
| ***Fish + Habitat*** |  |  |  |  |
| Freshwater fish | 5.7772 | <0.0001 | 0.2902 | 0.0033 |
| Marine fish | -0.1004 | 0.9200 | 0.3407 | 0.1010 |
| ***Supplemental types*** |  |  |  |  |
| Additives | 4.4438 | <0.0001 | 0.3736 | 0.0014 |
| Ingredients | 4.0509 | <0.0001 | 0.1659 | 0.1300 |
| ***Trophic level*** |  |  |  |  |
| Low trophic level species | 2.2907 | 0.0220 | 0.2396 | 0.0204 |
| Medium trophic level species | 4.7582 | <0.0001 | 0.2944 | 0.0178 |

**Table S7:** Outcomes of Egger’s regression test and Begg’s rank correlation test to evaluate publication bias during the study for condition factor comparisons.

| **Condition factor** | Egger’s regression test | | Begge’s rank correlation test | |
| --- | --- | --- | --- | --- |
|  | z value | p-value | Kendall's tau | p-value |
| All species | 1.2646 | 0.2060 | 0.0987 | 0.2458 |
| Subgroups |  |  |  |  |
| ***Species category*** |  |  |  |  |
| Fish species | -2.8478 | 0.0044 | -0.1438 | 0.1182 |
| Shrimps | 3.3971 | 0.0007 | 0.9444 | < 0.0001 |
| ***Fish + Habitat*** |  |  |  |  |
| Freshwater fish | -2.9205 | 0.0035 | -0.2074 | 0.0427 |
| Marine fish | 0.3085 | 0.7577 | 0.1556 | 0.6007 |
| ***Supplemental types*** |  |  |  |  |
| Additives | 0.0489 | 0.9610 | 0.0480 | 0.7507 |
| Ingredients | 1.5150 | 0.1298 | 0.0871 | 0.4249 |
| ***Trophic level*** |  |  |  |  |
| Low trophic level species | 3.5797 | 0.0003 | 0.2569 | 0.0911 |
| Medium trophic level species | 0.9128 | 0.3614 | 0.2286 | 0.0510 |
| High trophic level species | -0.3242 | 0.7457 | 0.1667 | 0.6672 |

**Table S8:** Outcomes of Egger’s regression test and Begg’s rank correlation test to evaluate publication bias during the study for hepatosomatic index comparisons.

| **Hepatosomatic index** | Egger’s regression test | | Begge’s rank correlation test | |
| --- | --- | --- | --- | --- |
|  | z value | p-value | Kendall's tau | p-value |
| All species | -2.4877 | 0.0129 | -0.2228 | 0.0657 |
| Subgroups |  |  |  |  |
| ***Species category*** |  |  |  |  |
| Fish species | -2.5239 | 0.0116 | -0.1724 | 0.1884 |
| Shrimps | -1.5452 | 0.1223 | -1.0000 | 0.0833 |
| ***Fish + Habitat*** |  |  |  |  |
| Freshwater fish | -2.4825 | 0.0130 | -0.2062 | 0.1470 |
| Marine fish | -0.3860 | 0.6995 | -0.3333 | 0.7500 |
| ***Supplemental types*** |  |  |  |  |
| Additives | -0.0245 | 0.9804 | 0.0909 | 0.7612 |
| Ingredients | -2.5951 | 0.0095 | -0.3123 | 0.0384 |
| ***Trophic level*** |  |  |  |  |
| Low trophic level species | -3.3114 | 0.0009 | -0.5273 | 0.0264 |
| Medium trophic level species | -1.2099 | 0.2263 | -0.2648 | 0.0812 |


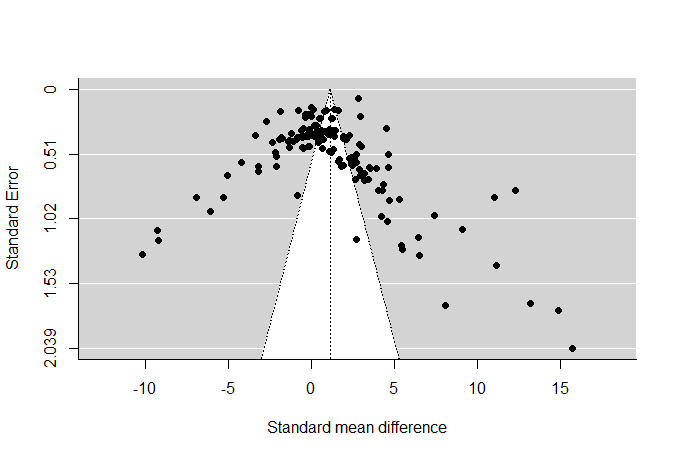
**Figure S1**. Evidence of publication (reporting) bias. (a) Funnel plot of standardized mean difference of final body weight (FBW); in the absence of bias the points should resemble a symmetrical inverted funnel. (b) Funnel plot showing the additional missing studies imputed by **trim and fill in white;** the white vertical line indicates the possible summary if the theoretical missing studies were to be included.

(a)


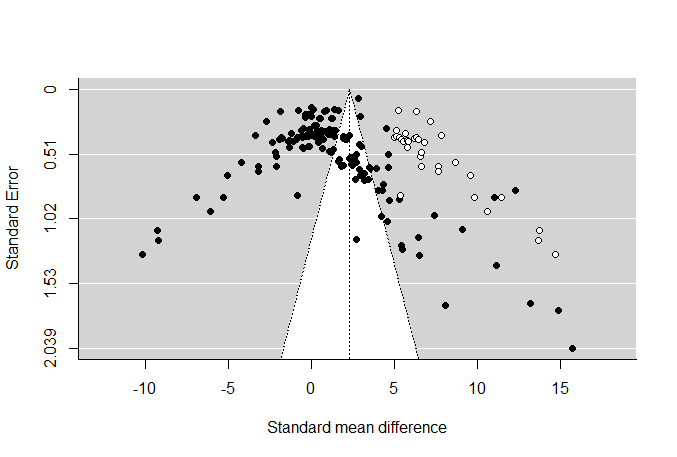


(b)

**Figure S2**. Evidence of publication (reporting) bias. (a) Funnel plot of standardized mean difference of specific growth rate (SGR); in the absence of bias the points should resemble a symmetrical inverted funnel. (b) Funnel plot showing the additional missing studies imputed by **trim and fill in white;** the white vertical line indicates the possible summary if the theoretical missing studies were to be included.


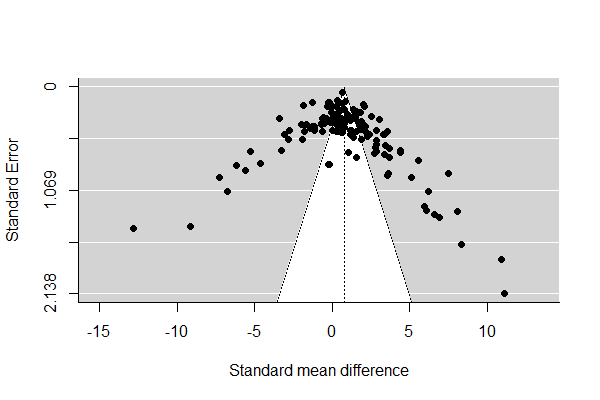


(a)


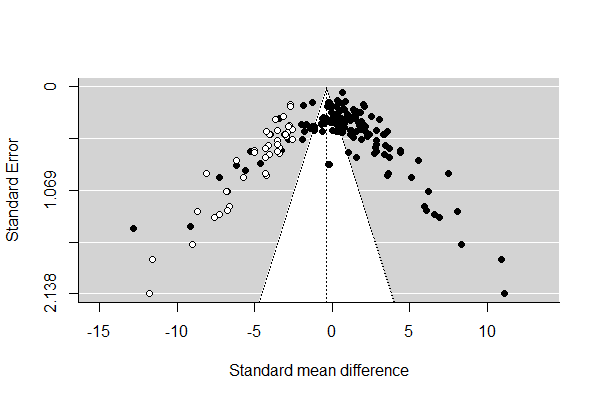


(b)

**Figure S3**. Evidence of publication (reporting) bias. (a) Funnel plot of standardized mean difference of feed conversion ratio (FCR); in the absence of bias the points should resemble a symmetrical inverted funnel. (b) Funnel plot showing the additional missing studies imputed by **trim and fill in white;** the white vertical line indicates the possible summary if the theoretical missing studies were to be included.


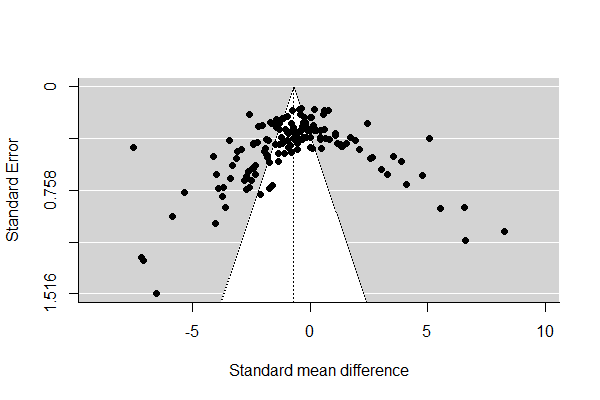


(a)


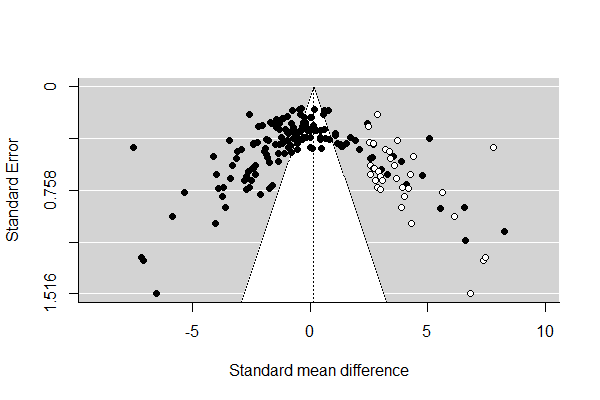


(b)

**Figure S4**. Evidence of publication (reporting) bias. (a) Funnel plot of standardized mean difference of protein efficiency ratio (PER); in the absence of bias the points should resemble a symmetrical inverted funnel. (b) Funnel plot showing the additional missing studies imputed by **trim and fill in white;** the white vertical line indicates the possible summary if the theoretical missing studies were to be included.


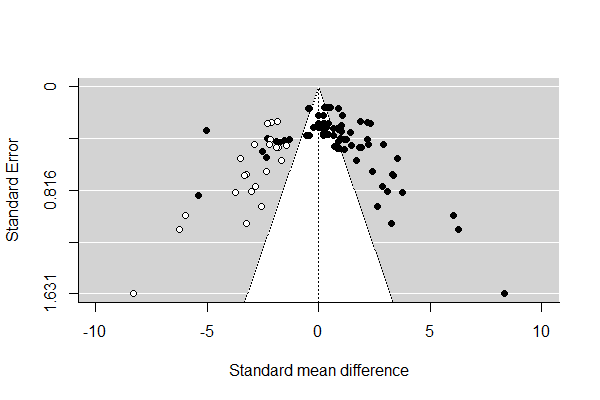

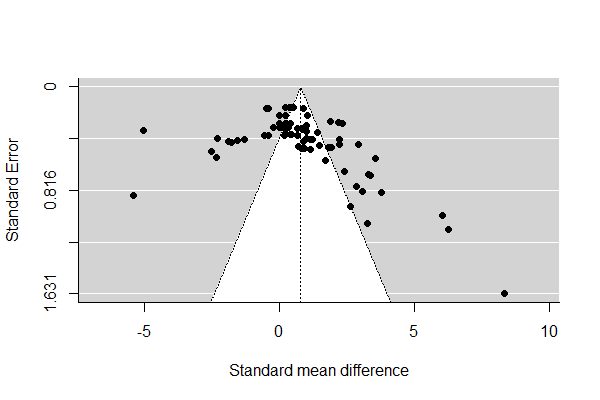


(b)

(a)

**Figure S5**. Evidence of publication (reporting) bias. (a) Funnel plot of standardized mean difference of condition factor (CF); in the absence of bias the points should resemble a symmetrical inverted funnel. (b) Funnel plot showing the additional missing studies imputed by **trim and fill in white;** the white vertical line indicates the possible summary if the theoretical missing studies were to be included.


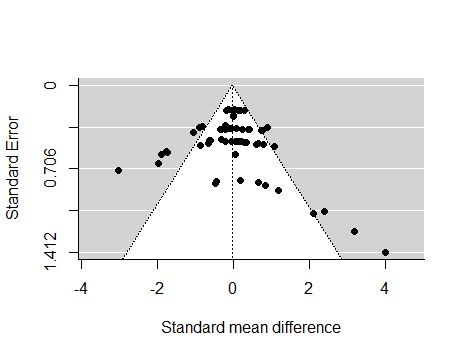


(a)


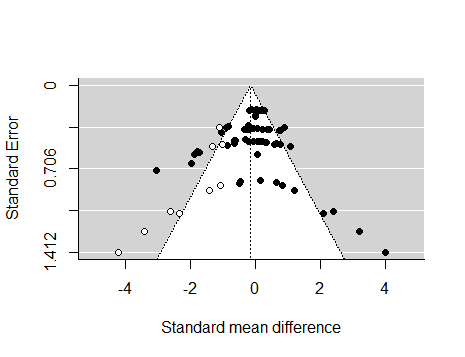


(b)

**Figure S6**. Evidence of publication (reporting) bias. (a) Funnel plot of standardized mean difference of hepatosomatic index (HSI); in the absence of bias the points should resemble a symmetrical inverted funnel. (b) Funnel plot showing the additional missing studies imputed by **trim and fill in white;** the white vertical line indicates the possible summary if the theoretical missing studies were to be included.


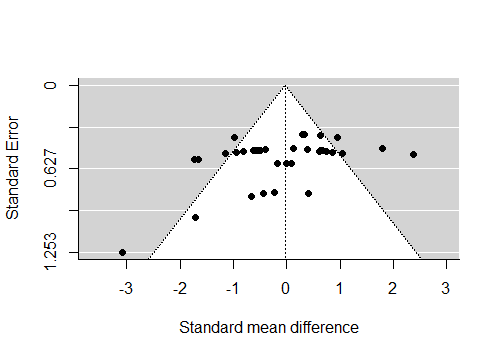


(a)


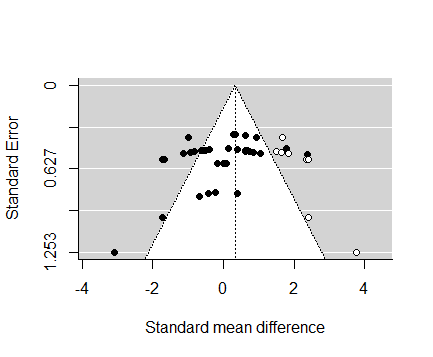


(b)

**Figure S7.** Hedges’ g comparisons for CF and HSI (mean ± 95% confidence interval), subgroup analysis (random-effect model). The confidence interval intersecting with the dashed line indicated no significant differences between the control group and treatment group, and vice versa.


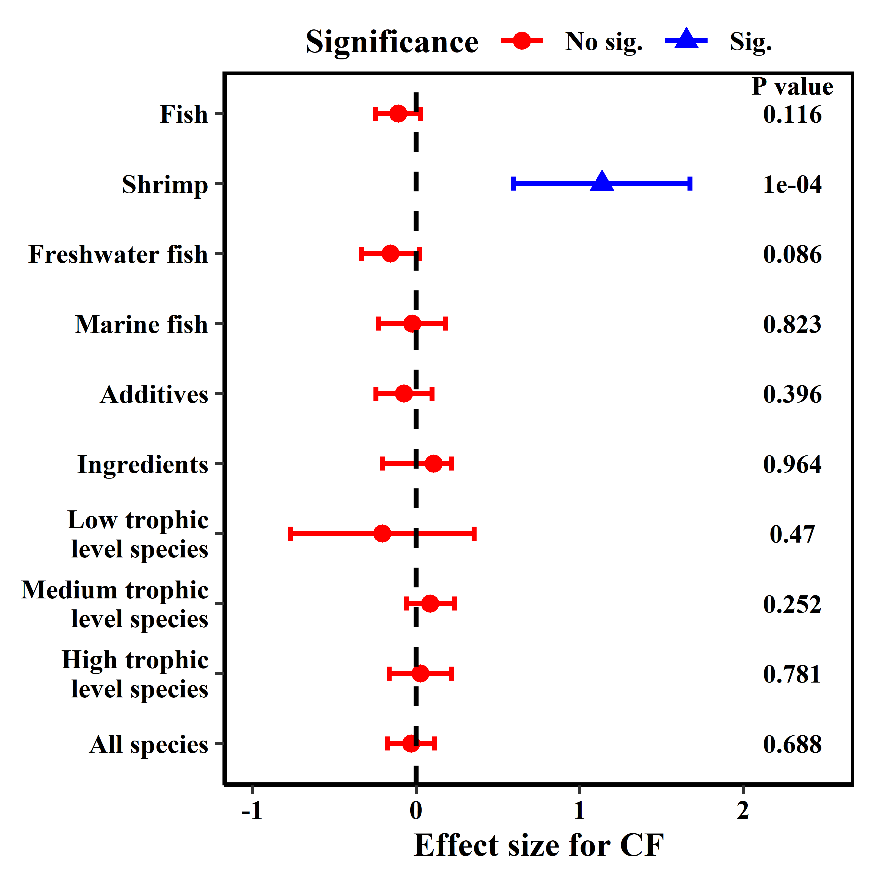

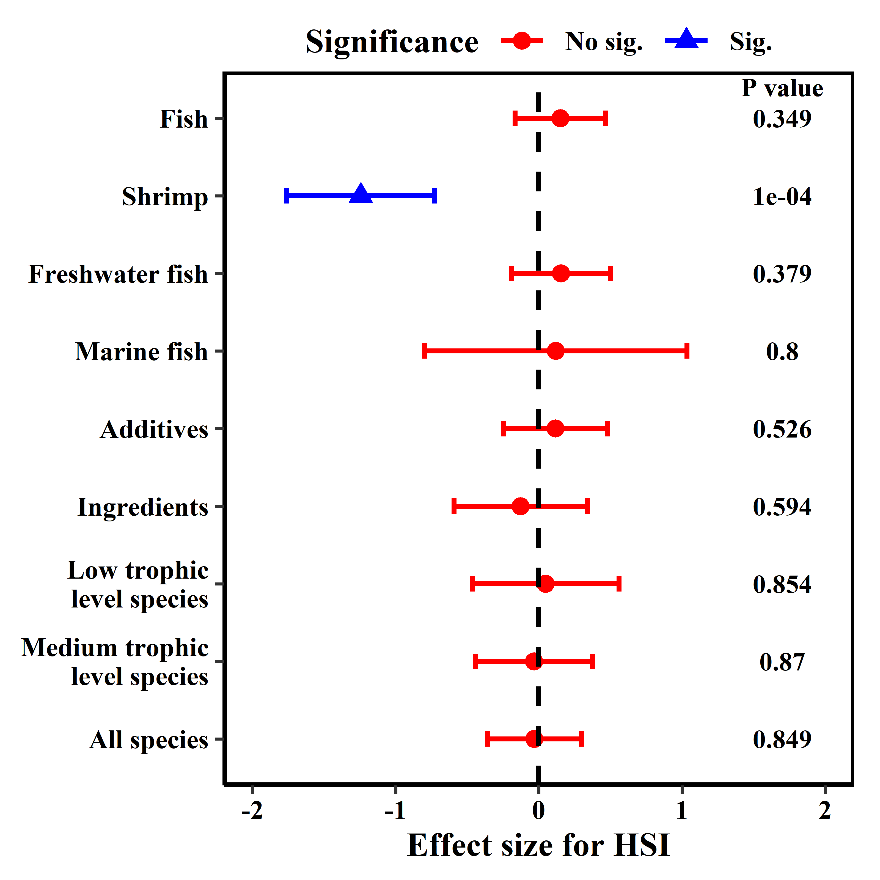


R code for FBW dataset analysis

### For Hedge’s g calculation

datum <- read.csv(file.choose())

library(metafor)

datum1 <- escalc (measure = "SMD", n1i = nti, m1i = mti, sd1i = sdti, n2i = nci, m2i = mci, sd2i = sdci, data = datum) ## calculate yi and vi (SMD)

write.csv(datum1,file = "FBW SDM 20220415.csv")

##pooled effect size and sensitivity analysis

datum <- read.csv(file.choose())

res.FBW <- rma(yi=yi,vi=vi,data=datum,method="REML")

summary(res.FBW)

inf.FBW <- influence(res.FBW)

plot(inf.FBW)

baujat(res.FBW)

forest(res.FBW,slab = paste(datum$Name))

funnel(res.FBW,xlab = "Standard mean difference")

help("trimfill")

tlf.FBW <- trimfill(res.FBW,side = "right")

tlf.FBW

funnel(tlf.FBW, xlab = "Standard mean difference")

tlf.FBW <- trimfill(res.FBW,side = "left")

tlf.FBW

funnel(tlf.FBW, xlab = "Standard mean difference")

regtest(res.FBW) ##Egger's test

ranktest(res.FBW) ##Begge's test

leave1out(res.FBW)

summary(leave1out(res.FBW))

##subgroup analysis (fish and shrimp)

res.FBW.fish <- rma(yi=yi,vi=vi,data = datum, subset=(Species.category=="fish"),method = "REML")

summary(res.FBW.fish)

res.FBW.shrimp <- rma(yi=yi,vi=vi,data = datum, subset=(Species.category=="shrimp"),method = "REML")

summary(res.FBW.shrimp)

regtest(res.FBW.fish) ##Egger's test

regtest(res.FBW.shrimp) ##Egger's test

ranktest(res.FBW.fish) ##Begge's test

ranktest(res.FBW.shrimp) ##Begge's test

### forest plot

dataset <- read.csv(file.choose())

dataset

dataset$Subgroups <- factor(dataset$Subgroups,levels = c("All species","Medium trophic level species","Low trophic level species","Ingredients","Additives","Marine fish","Freshwater fish","Shrimp","Fish")) ##

dataset$Subgroups

library(ggplot2)

library(stringr)

shapes <- c("Sig" = 19,"No sig." = 19)

label <- dataset[,7]

label

x <- c(5.7,5.7,5.7,5.7,5.7,5.7,5.7,5.7,5.7,5.7)

y <-c(10,9,8,7,6,5,4,3,2,1)

label1 <- c("P value")

x1 <- c(5.5)

y1 <- c(10.4)

p <- ggplot(dataset,aes(Mean,Subgroups,colour = Significance, shape = Significance)) +

geom_point(size = 2.5) +

scale_color_manual(values = c("red","blue")) +

geom_errorbarh(aes(xmax =Upper, xmin = Lower), height = 0.2,cex = 0.8) +

scale_x_continuous(limits= c(-3, 6), breaks= seq(-3, 6, 1)) +

geom_vline(aes(xintercept = 0),linetype = 8,cex = 0.8) +

xlab("Effect size for FBW") + ylab('') +

geom_text(aes(x=x,y=y,label = label),size = 3,color = "black", family = 'serif',fontface = "bold") +

geom_text(aes(x=x1,y=y1,label = label1),size = 3,color = "black", family = 'serif',fontface = "bold") +

theme_classic() +

theme(panel.border = element_rect(fill = NA,color = "black",size = 1, linetype = 1)) +

theme(text = element_text(family='serif', face = "bold",color = "black")) +

theme(axis.text.x = element_text(color = "black")) +

theme(axis.text.y = element_text(color = "black")) +

scale_y_discrete(labels = function(x) str_wrap(x,width = 15)) +

theme(plot.margin = unit (c(0.5,0.3,0.2,-0.2),"cm"), legend.position = "top", legend.margin = margin(0.5,0.5,0.5,0.5),legend.box.margin = margin(-10,-10,-10,-10))

p

ggsave("FBW forest plot.tiff",width = 4, height = 4, dpi = 800)

##meta-regression for additive

library(ggplot2)

library(mgcv)

library(segmented)

dataset <- read.csv(file.choose())

p <- ggplot(dataset,aes(Inclusion,yi)) + geom_point() + geom_smooth(span = 0.8)

p

lm_FBW <- lm(yi~Inclusion,data=dataset) ##

lm_seg1 <- segmented(lm_FBW,seg.Z = ~Inclusion,npsi=2)

summary(lm_seg1)

dataset <- read.csv(file.choose())

dataset_additive <- subset(dataset,dataset$Ingredients == "additive")

dataset_additive

d <- dataset_additive[1:64,]

d

library(ggplot2)

library(mgcv)

install.packages("maps")

library(maps)

p <- ggplot(dataset_additive,aes(x = Inclusion, y=yi, colour = Species.cat)) +

geom_point(alpha = 0.5,size = 3) +

scale_color_manual(values = c("red","blue")) +

theme_bw() +

theme(legend.position = "top") +

theme(legend.title=element_blank()) +

theme(panel.grid.major = element_blank(),panel.grid.minor = element_blank())

p

p1 <- p + geom_smooth(data=dataset_additive[1:64,],aes(x=Inclusion, y = yi),method="loess",se = FALSE, color="red",span = 0.8)

p1 <- p + geom_smooth(data=dataset_additive[1:64,],aes(x=Inclusion, y = yi),method="lm", se = FALSE,colour="#FF6600",formula = y ~ x + I(x^2),size = 0.8)

p1

ggsave("FBW regression.tiff",width = 4, height = 4, dpi = 800)

p2 <- p1 + geom_smooth(data=dataset_additive[65:78,],aes(x=Inclusion, y = yi),method="lm", se = FALSE,colour="#3366FF",formula = y ~ x + I(x^3), size = 0.8)

p2

ggsave("regression plot.tiff",width = 4, height = 4, dpi = 800)

p3 <- p2 + geom_segment(aes(x=1.4575,y=2.3071,xend = 1.4575,yend = -10),lty = 1,colour = "black", arrow = arrow(length = unit(0.2,"cm"))) +

geom_hline(aes(yintercept = 0),linetype = 8,cex = 0.5)+

theme(panel.border = element_rect(fill = NA,color = "black",size = 1, linetype = 1)) +

theme(text = element_text(family='serif', face = "bold",color = "black")) +

theme(axis.text.x = element_text(color = "black",size = 10)) +

theme(axis.text.y = element_text(color = "black", size = 10)) +

labs(x = "Spirulina inclusion level (%)",y = "Effect size for FBW") +

theme(axis.title.y = element_text(margin = margin(0,-0.1,0,0,"cm"))) +

scale_y_continuous(limits= c(-10, 15), breaks= seq(-10, 15, 5)) +

theme(plot.margin = unit (c(0.5,0.3,0.2,0.3),"cm"), legend.margin = margin(0.5,0.5,0.5,0.5),legend.box.margin = margin(-10,-10,-10,-10))

p3

p4 <- p3 + annotate("text",x=2,y=-9.5,label = "x = 1.458",family="serif",colour = "black",fontface = "bold")

p4

ggsave("regression FBW.tiff",width = 4, height = 5 , dpi = 800)

help("arrows")

dataset_additive_fish <- subset(dataset_additive, dataset_additive$Species.cat == "Fish")

dataset_additive_fish

fitlm <- lm(yi ~ Inclusion + I(Inclusion^2),data = dataset_additive_fish)

summary(fitlm)

library(segmented)

lm_seg <- segmented(fitlm,seg.Z = ~Inclusion,npsi = 2)

summary(lm_seg)

dataset_additive_shrimp <- subset(dataset_additive, dataset_additive$Species.cat == "Shrimp")

dataset_additive_shrimp

fitlm1 <- lm(yi ~ Inclusion + I(Inclusion^3),data = dataset_additive_shrimp)

summary(fitlm1)

library(segmented)

lm_seg <- segmented(fitlm,seg.Z = ~Inclusion,npsi = )

summary(lm_seg)

##regression for protein source

dataset <- read.csv(file.choose())

dataset_ingredient <- subset(dataset,dataset$Ingredients == "ingredient")

dataset_ingredient

library(ggplot2)

library(mgcv)

library(maps)

p <- ggplot(dataset_ingredient,aes(Inclusion,yi,colour = x)) +

geom_point() + geom_smooth(span = 0.8)

p

dataset_ingredient_Fish <-subset(dataset_ingredient,dataset_ingredient$x == "Fish")

dataset_ingredient_Fish

fitlm3 <- lm(yi ~ Inclusion + I(Inclusion^2),data = dataset_ingredient_Fish)

summary(fitlm3)

library(segmented)

lm_seg3 <- segmented(fitlm3,seg.Z = ~Inclusion,npsi = 2)

summary(lm_seg3)

dataset_ingredient_Shrimp <-subset(dataset_ingredient,dataset_ingredient$x == "Shrimp")

dataset_ingredient_Shrimp

fitlm4 <- lm(yi ~ Inclusion,data = dataset_ingredient_Shrimp)

summary(fitlm4)

library(segmented)

lm_seg4 <- segmented(fitlm4,seg.Z = ~Inclusion,psi = 15,npsi = 1)

summary(lm_seg4)

help(scale_size)

p <- ggplot(dataset_ingredient,aes(x = Inclusion, y=yi, colour = x,size = FMd)) +

geom_point(alpha = 0.5) +

scale_color_manual(values = c("red","blue")) +

theme_bw() +

theme(legend.position = "top") +

scale_size(range = c(1,5),breaks=c(40,20,10,5)) +

theme(panel.grid.major = element_blank(),panel.grid.minor = element_blank())

p

p1 <- p + geom_smooth(data=dataset_ingredient[1:60,],aes(x=Inclusion, y = yi),method="lm", se = FALSE,colour="#FF6600",formula = y ~ x + I(x^2),size = 0.8)

p1

p2 <- p1 + geom_segment(aes(x=5,y=11.261,xend = 10.961,yend = 0.733874),lty = 1,colour = "#3366FF",size = 0.8)

p2

p3 <- p2 + geom_segment(aes(x=10.961,y=0.733874,xend = 40,yend = -4.300812),lty = 1,colour = "#3366FF",size = 0.8)

p3

p4 <- p3 + geom_hline(aes(yintercept = 0),linetype = 8,cex = 0.5) +

geom_segment(aes(x=24.5245,y=0,xend = 24.5245,yend = -10),lty = 1,colour = "black", size=0.5, arrow = arrow(length = unit(0.2,"cm"))) +

annotate("text",x=31.5,y=-9.5,label = "x = 24.53",family="serif",colour = "#FF6600",fontface = "bold") +

geom_segment(aes(x=14.944,y=0,xend = 14.944,yend = -12),lty = 1,colour = "black", size=0.5, arrow = arrow(length = unit(0.2,"cm"))) +

annotate("text",x=8.0,y=-11.5,label = "x = 14.95",family="serif",colour = "#3366FF",fontface = "bold") +

geom_segment(aes(x=10.96,y=0.7339,xend = 10.96,yend = -6),lty = 1,colour = "black", size=0.5, arrow = arrow(length = unit(0.2,"cm"))) +

annotate("text",x=8.5,y=-6.8,label = "x = 10.96",family="serif",colour = "#3366FF",fontface = "bold")+

theme(panel.border = element_rect(fill = NA,color = "black",size = 1, linetype = 1)) +

theme(text = element_text(family='serif', face = "bold",color = "black")) +

theme(axis.text.x = element_text(color = "black",size = 10)) +

theme(axis.text.y = element_text(color = "black", size = 10)) +

labs(x = "Spirulina inclusion level (%)",y = "Effect size for FBW") +

theme(axis.title.y = element_text(margin = margin(0,-0.1,0,0,"cm"))) +

scale_y_continuous(limits= c(-12, 16), breaks= seq(-12, 16, 6)) +

scale_x_continuous(limits= c(0, 60), breaks= seq(0, 60, 10)) +

theme(plot.margin = unit (c(0.5,0.3,0.2,0.3),"cm"), legend.margin = margin(0.5,12,0.5,0.5),legend.box.margin = margin(-10,-10,-10,-10),legend.spacing.x = unit(0.0001,'cm')) +

guides(colour=guide_legend(override.aes = list(size=3)))

p4

ggsave("replacement level FBW.tiff",width = 4, height = 5 , dpi = 800)
